# Multiple uses of app instead of using multiple apps – a case for rethinking the digital health technology toolbox

**DOI:** 10.1017/S2045796020000013

**Published:** 2020-01-31

**Authors:** John Torous, Aditya Vaidyam

**Affiliations:** Division of Digital Psychiatry, Beth Israel Deaconess Medical Center, Harvard Medical School, Boston, MA, USA

**Keywords:** Depression, health outcomes, mental health, other psychosocial techniques/treatments

## Abstract

There are tens of thousands of mental health-related apps available today – representing extreme duplication in this digital age. Instead of a plethora of apps, there is a need for a few that meet the needs of many. Focusing on transparency and free sharing of software, we argue that a collaborative approach towards apps can advance care through creating customisable and future proofed digital tools that allow all stakeholders to engage in their design and use.

Form follows function is a principle first associated with architectural design and now software architecture. The shape and layout of building will determine the function of the space inside and the database and middleware of a computer program will determine what functions it can perform. Form follows function is relevant in digital health as well, and can explain why most health apps fail and yet one may still succeed.

mindLAMP is an open source and freely sharable health app that our team and network of collaborators created to offer the core functions that users expect from digital health tools like education, innovative assessments, digital phenotyping, self-management tools and connections to human support. To date, over 1000 people have used it in research studies and our division uses the app to augment care in our ‘digital clinic’ offered in Boston. You can learn more about mindLAMP by visiting digitalpsych.org/lamp/about. But behind the visible functions such as mood tracking or peer chat is a unique architecture that allows the app to be flexible, adaptable and customisable to the unique clinical needs, preferences, languages and visualisation requests of each user. By carefully designing not only the app functionality but also the platform to support it, we argue that a single app can offer clear benefits outweighing a sea of individual apps.

The lack of engagement with individual mental health apps, with recent data suggesting a 4% daily open rate (Baumel *et al*., 2019), highlights the challenges for even those apps featured highly on the commercial marketplaces. Reasons for the lack of engagement with these apps vary but include a lack of customisation (Fleming *et al*., 2019), skepticism of efficacy (Muse and Topol, 2019), concerns around privacy (Huckvale *et al*., 2019), challenges around usability (Sarkar *et al*., 2016), incompatibility with older or cheaper phones and lack of actionability (Torous *et al*., 2018). There are thousands of apps that offer functions such as mood tracking or mindfulness – but those numbers decay to single digits when looking for safe, evidence-based and adaptable tools. They approach zero when also demanding compatibility with older phones, working with poor internet connectivity, and being easy to use for those who are not already possessing high technology literacy. It is not for the lack of function that these myriad apps fail, but for the lack of form and the right support behind them and their screens.

While a single app may seem like an anachronism in the year 2020, a single app supported by the right form, in this case a platform, offers a solution. We designed mindLAMP with many use cases in mind driven by collaboration with diverse stakeholders including numerous patients (Torous *et al*., 2019). One often discussed yet rarely designed for group of users is those people with older or cheaper smartphones, restricted access to wifi, limited data plans and low technology literacy. Our team's conversations with clinicians and patients in rural India, remote villages in Nepal and the community mental health centres across the United States underscored for how many people apps today do not work for. Thus, we sought to create a single app, supported by the right platform to offer the same utility and value to users whether they be located in Bhopal or Boston.

Focusing on the needs of many users from many backgrounds instead of a single function of the app led us to seven design objectives: (1) global impact, (2) open source, (3) simplicity, (4) efficiency, (5) ethical design, (6) security and (7) privacy. Appendix 1 offers architectural design and infrastructure implementation details for mindLAMP and how we sought to ensure the form of the platform supports its functions. We operationalised these objectives through framing the app around data standards and accessible technology as pathway towards offering the same functions for diverse users.

While representative, structured and secure data are not a feature on the mind of a user when selecting a mental health app – it is the foundation of any app. Without representative data to capture the live experience of a user be it through surveys or cognitive tests, step count or heart rate variability, the entire premise of any app is faulty as it would have collected incorrect data. Without structured data to support analysis and actionability of the data, for example triggering customisable just-in-time-adaptive interventions, app data will be less meaningful. Finally, without security measures in place to protect privacy and enable secure sharing, data will be a liability and even source of potential harm. By focusing on these features in the early stages of mindLAMP, we offer a form that can support diverse uses today and new ones tomorrow. For example, one user utilises the app to alert her to drink water as her step count reaches certain pre-set thresholds throughout the day in order to track her mood in response to more physical activity. At the time of this writing, we are aware of a few hybrid hydration and mental health apps yet today able to offer such functionality to that person.

The second complementary component, straightforward and accessible technology ensures the app is available to as many people as possible. No two patients have the same needs and thus the ability to customise app use is critical. Customising the app can mean meeting with a clinician or digital navigator (Noel *et al*., 2019) or setting up the technology without any guidance. Regardless of the method, the investment in helping shape the final product will likely fuel engagement, especially compared to an off the shelf and ready to go app. Ensuring the functions of the app remain accessible means ensuring those with the least resources – and often the oldest phones and least ability to access the internet – can still benefit. In creating mindLAMP we have focused on low power and connectivity support so that a user in rural Nepal can still access the majority of functions even if they cannot always access the internet. This means a small version of the mindLAMP server runs on each device the app is installed on – even a smartwatch – so that there is the right support to keep the core functions running in almost any situation. Simplifying the app to be usable by all is an ongoing process that improves with user feedback, and offering support for multiple languages as well as pictorial icons has enabled mindLAMP to currently engage users from Shanghai in China to Tijuana in Mexico. At the time of this writing, we are aware of a few apps offering multi-language mental health functions.

Returning the challenges of many mental health apps today as outlined in paragraph two, further advantages of a single app approach become clear. Concerns around privacy are always warranted, but assessing and understanding the risks of one app are a much simpler task than that for a toolkit. The ability to customise and create unique interventions triggered by data, or just a simpler alarm, offers further incentives for use. Continued learning from global users ensures usability and technical compatibility issues can be quickly addressed. A less concrete but equally important advantage is the ability to integrate apps into care via the single app approach. Drawing from our clinical experience of one patient who (Sandoval *et al*., 2017) created his own toolkit of mental health apps for therapy, that panoply of apps on his phone soon became a barrier to care. The messages from these numerous apps often conflicted (‘In this moment relax’, ‘In this moment challenge yourself’, ‘In this moment contact a friend’) and left the patient unsure and our team equally confused. In an era where integrated care is the goal, apps risk fragmenting care and soiling data if not access to data and results is not simple to access and share.

A single app approach can be powerful but also presents challenges. The ultimate success of the mindLAMP app and platform behind it must be its broad adoption and expansion by others. While most health apps hide their foundation as propriety trade secrets, we openly offer ours towards forming a community of collaborators. The representative, structured and secure data as well as straightforward and accessible technology approaches are only as powerful as they are adopted by others to evolve and grow mindLAMP. Simply put, a single app can serve multiple purposes and meet the demands of many users only if has the support, input and upkeep of many.

The mindLAMP project continues to expand and we encourage readers to explore our blueprint in Appendix 1, code shared online at gitub and to trial and customise the app itself at digitalpsych.org/lamp/about. We realise that with hundreds of thousands of health apps ready to download today it is possible to build an impressive toolkit of diverse apps. But, even the best toolkit is only as useful as those tools are utilised. With mindLAMP as a single app we believe that the focus does not need to be on tools but rather the people, using a tool, towards recovery.

## Supplementary material

For supplementary material accompanying this paper visit http://dx.doi.org/10.1017/S2045796020000013. click here to view supplementary material
