# Supplementary material for: Multiple uses of app instead of using multiple apps – a case for rethinking the digital health technology toolbox
Source: Epidemiol Psychiatr Sci. 2020 Jan 31;29:e100. doi: 10.1017/S2045796020000013 (PMC7216034; doi:10.1017/S2045796020000013)
Supplement: Supplementary file 1 [file S2045796020000013sup001.pdf]

The most current version of this document shall be made available at: https://docs.lamp.digital/ and https://digitalpsych.org/ **[2020-01-01]**

The **LAMP Platform** Reference

Architectural design & **Infrastructure** implementation.

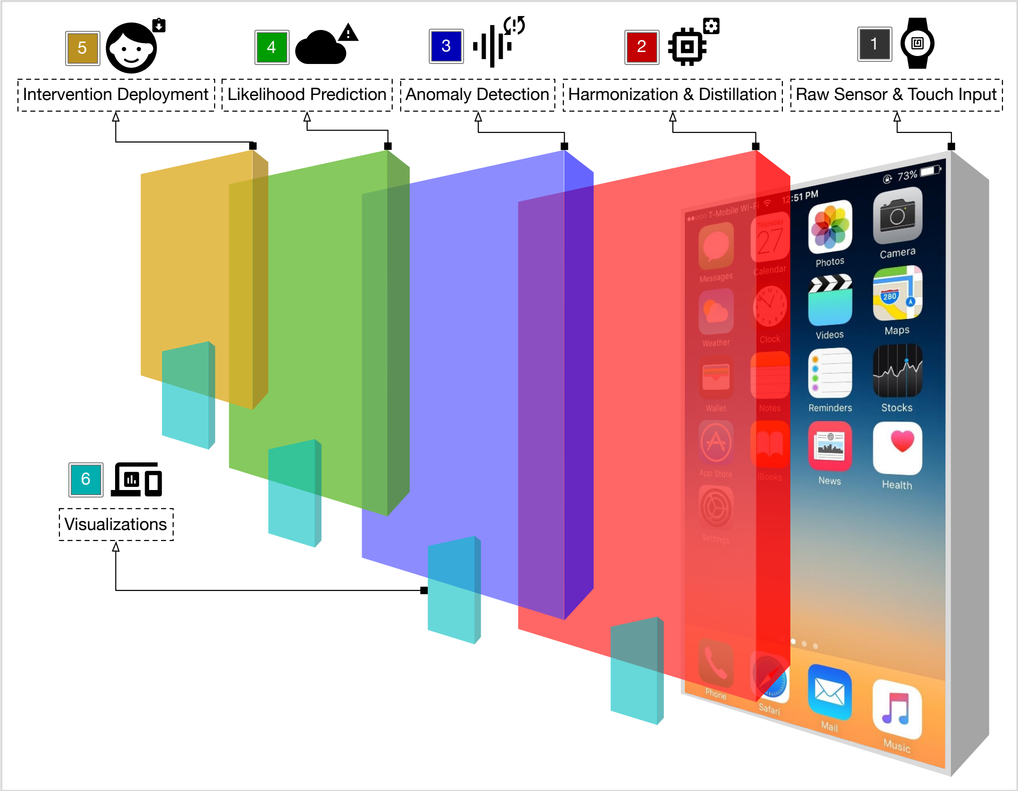

TABLE OF CONTENTS

TABLE OF CONTENTS....2

PROJECT SCOPE 4

STAKEHOLDERS 4
DESIGN OBJECTIVES 4
ENGINEERING PRINCIPLES 5

PLATFORM OVERVIEW 6

PLATFORM OVERVIEW 6
STEP 1: SENSOR EVENTS ARE RECORDED IN REAL-TIME 7
STEP 2: COLLECTED EVENTS ARE CACHED, AWAITING SERVER REACHABILITY. 7
STEP 3: SERVER RECEIVES AND PROCESSES REQUESTS FROM THE APP. 7
STEP 4: SERVER COORDINATES INTERNAL COMPONENTS FOR PROCESSING. 8
STEP 5: DATABASE RECORDS THE INCOMING DATA. 8
STEP 6: THE SCHEDULER COORDINATES AND RUNS AUTOMATIONS. 8
STEP 7: APPLETS ARE LAUNCHED INTO A SAFE ENVIRONMENT AND RUN. 9
STEP 8: APPLET RESULTS ARE SAVED AFTER BEING RUN. 9
STEP 9: APPLET RESULTS ARE PERSISTED FOR LATER ACCESS. 9
STEP 10: SCHEDULER UPDATES INVOCATION AND AUDIT LOG. 10
STEP 11: REQUEST COMPLETES WITH ANY RESPONSE DATA. 10

LOW POWER & CONNECTIVITY SUPPORT 11

LIMITATIONS & STRATEGIES 13

CONTINUOUS MONITORING & INTERVENTION DELIVERY 14

COMPONENTS 15

LAMP PROTOCOL 15
BOUNDARIES 19
LAMP SERVER 21
MINDLAMP APP 23
MINDLAMP WATCH COMPLICATION 24
MINDLAMP DASHBOARD 25
ACTIVITIES 37
SENSORS 39

CLOUD DEPLOYMENT & INFRASTRUCTURE 40

PROVISIONING CLOUD RESOURCES 40

THE AUTOMATIONS FRAMEWORK 42

TAGS AS ARBITRARY DATA ON RESOURCES 43
DATA-URI STRINGS IN TAGS 43
ATOMIC INDEXED ACCESS AND MODIFICATION 43

AUTOMATIONS AS MULTIDIMENSIONAL PLANES OF DATA WITHIN TAGS........ 44
FEDERATED SYSTEMS USING THE AUTOMATION FRAMEWORK........ 45

PROJECT SCOPE

As we aim to build a robust platform able to serve the needs of many individuals, we identify the following as key stakeholders whose perspectives and requirements are specifically considered within this document. Furthermore, we recognize a core set of goals that guide the process of design and development of this platform for use in research and in real world clinical settings.

Stakeholders

- **Patients**: individuals seeking increased quality of care under the guidance of a Clinician in a clinical healthcare setting and through the use of the platform.
- **Participants**: individuals seeking to advance scientific research in partnership with Researchers through enrollment in studies augmented by the of the platform.
- **Clinicians**: individuals seeking to directly provide care for Patients augmented by the use of the platform.
- **Researchers**: individuals seeking to advance scientific research through the use of the platform, in partnership with Participants.
- **Data Scientists**: individuals seeking to advance scientific research by architecting solutions and analyzing data collected by the platform, in partnership with Patients, Participants, Clinicians, or Researchers.
- **Developers**: individuals seeking to design and develop tools and applications for the platform to further the goals and meet the needs of Patients, Participants, Clinicians, Researchers, and Data Scientists.

Design Objectives

- **Global Impact**: we aim to design the platform for the Stakeholders of a global age, who come from different cultures, locations, and contexts. Both researchers and clinicians around the world should be able to understand, use, apply, and conduct research alongside the Platform. Patients and participants should be able to access and benefit from the Platform regardless of their environment.
- **Open Source**: we aim to develop a welcoming community around the platform that encourages interdisciplinary collaboration on a global scale, and offers the platform free of charge to those seeking to use it.
- **Simplicity**: we aim to design a platform whose components and total aggregate are inherently simple to understand, use, and apply to various engineering, research, or clinical contexts without excessive effort or technical support.
- **Efficiency**: we aim to design a platform whose facilities are built with efficiency and reliability in mind, such that low maintenance or computing overheads are incurred.
- **Ethical Design**: we aim to design a platform with ethically sound principles surrounding the use and collection of data, and allowing those who contribute data to maintain ownership of such data.
- **Security**: we aim to design systems using modern encryption methods that secure and maintain the integrity of any and all data both during transmission from one point to another and its storage, requiring consent and permission before one party is allowed to access another party's data, irrespective of the physical ownership of the hardware upon which the platform is operated.

- **Privacy**: we aim to design systems whose data storage inherently avoids and discourages personally identifiable information, though able to empower the originator of any piece of data with the right of ownership and consent, with HIPAA-compliant permanent non-recoverable deletion of data upon request.

Engineering Principles

Clinical needs and goals are first surveyed and understood, before they are translated into an engineering context for the features or technologies that need to be defined. Three core areas of needs and goals are modeled: (1) Data, (2) Network, and (3) Clinical, for the definition of core features and engineering strategies, as indicated in the figure below.

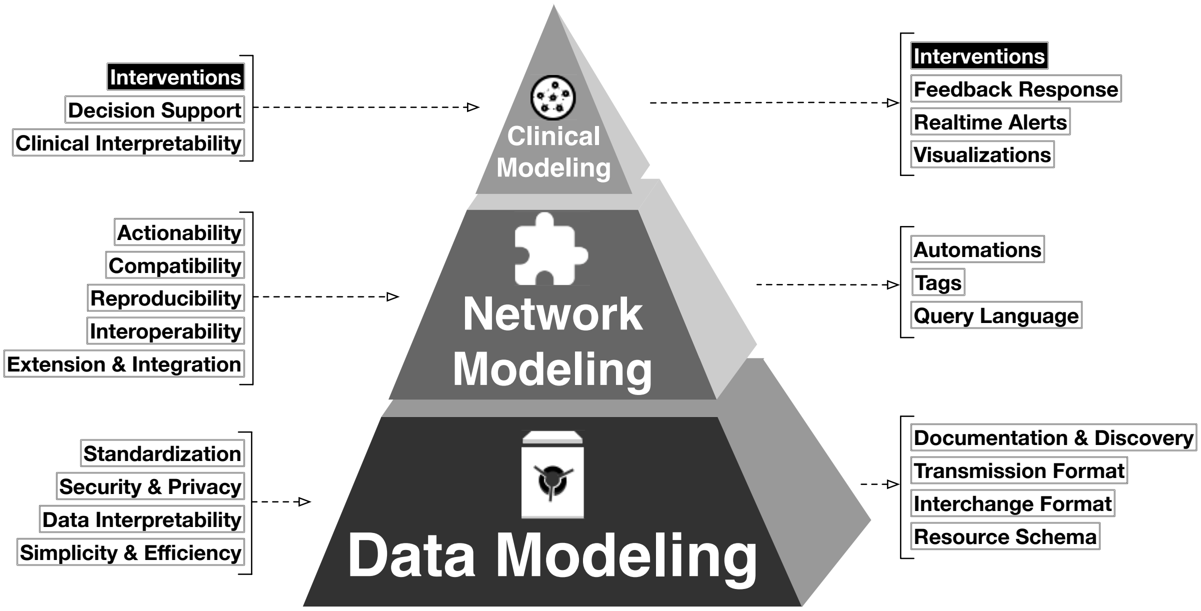

Terminologies in this document, such as the names of components, tools, frameworks, or otherwise, when initially introduced in text will be bolded and referred to as such in all later text.

We aim to both describe an abstract implementation-agnostic platform suitable for adaptation to any development requirement as well as to provide a reference implementation abiding by the guidelines set forth in this document.

As you read this documentation, you will see engineering suggestions or limitations presented in blocks such as this one highlighting current limitations and possible future development venues that underlie the design and architecture of the LAMP platform.

We aim to provide the same features and tools to clinicians, researchers, patients, and participants regardless of environment. Whether they have constant high-speed internet connectivity, or sparse and inconsistent internet connectivity, the LAMP platform will look, feel, and operate the same way, abstracting, to its best ability, the inconsistencies one may face in its day-to-day use.

As you read this documentation, you'll see clinical insight presented in blocks such as this one highlighting the real-world cases that inspire the design and architecture of the LAMP platform.

PLATFORM OVERVIEW

Platform Overview

The **LAMP Platform** consists of two broad domains: **(1) local**: the components that users will see and interact with through smartphone or wearable devices, and **(2) remote**: the components located off-device that process data, coordinate applets, and handle synchronization. The app serves to both capture diverse streams of sensor and activity measurements ranging from heart rate to mood and to prompt suggested user interactions. It informs the **LAMP Platform** with a micro-temporal slice of the user's physical and mental health. The server components supporting the **LAMP Platform** play an equally important role in securely encrypting and processing the data, establishing the user's "digital fingerprint" and predicting changes that could potentially result in relapse. app-based interventions can be deployed to improve the user's health and relevant health data can automatically be shared with authorized care team members.

The figures below detail one operation typically performed by the **LAMP Platform**. Shown on the left-hand side is the app, and on the right-hand side is the server. Note that both pieces consist of numerous components that work together as a modular distributed system to transfer and process data in a clinically relevant context. A full specification of all components and their interactivity is documented in later sections. Please note that an important distinction in naming is made between the **local** and **remote** domains: the components of the former are prefixed with "**mindLAMP**" where the components of the latter are prefixed only with "**LAMP**" as this distinction clarifies the scope and requirements of the components themselves.

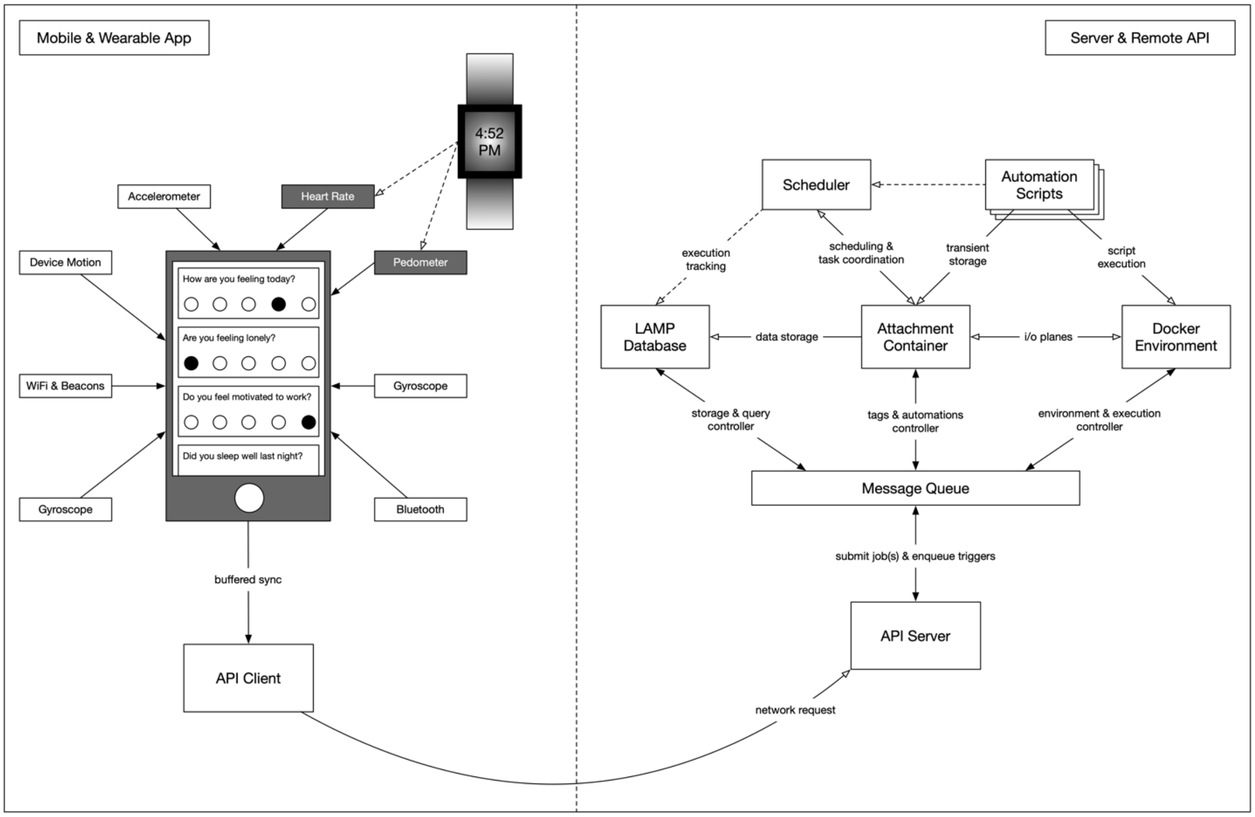

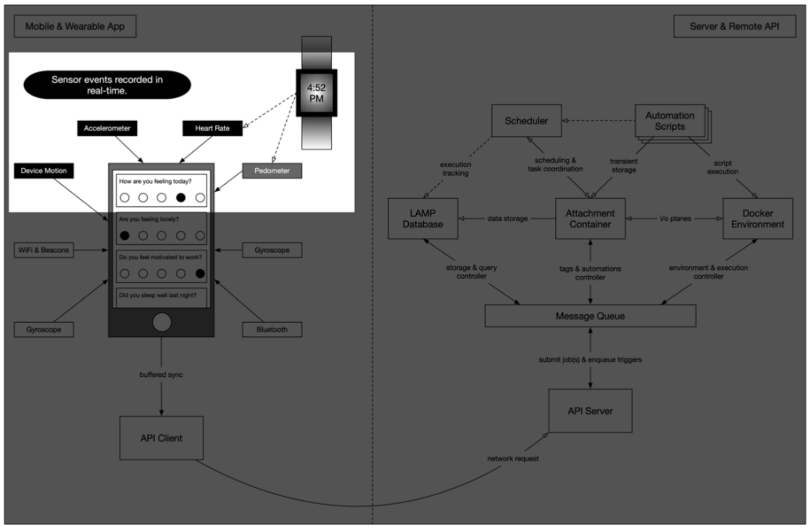

Step 1: Sensor events are recorded in real-time.

High-frequency sensors on the mobile or wearable device record measurements based on the user's current configuration settings defined by an administrator. This data is stored in a buffer on the device's hardware managed by the operating system (either iOS or Android) and provided to the app periodically while it is in the background.

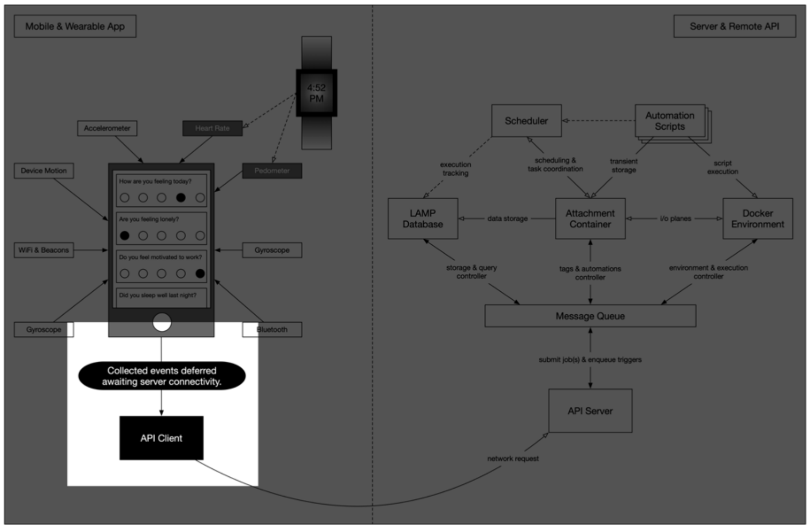

Step 2: Collected events are cached, awaiting server reachability.

The device's buffer and operating system make no guarantees to save data from the current moment for the next time the app is run in the background. Because of this and the likelihood that the remote server may not be reachable due to poor signal, the measurements are immediately cached by the app whenever it is notified in the background. When battery levels are sufficient, and network connectivity to the remote server available, and enough data is cached, the app begins synchronization.

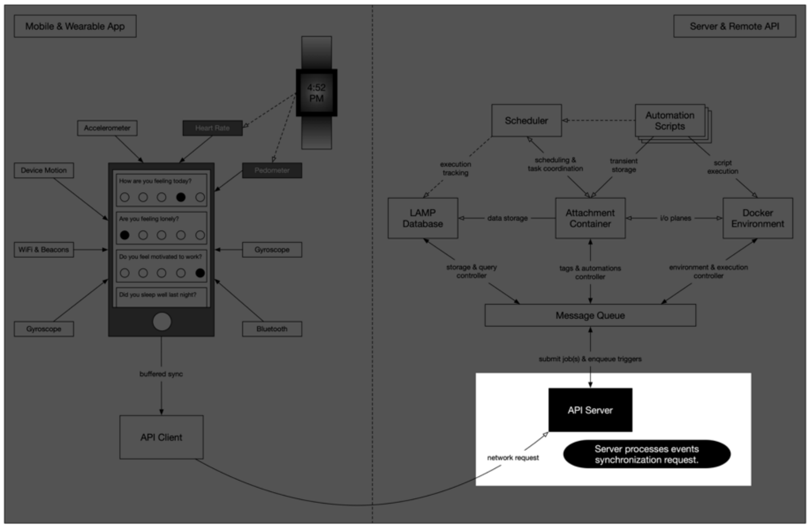

Step 3: Server receives and processes requests from the app.

The app submits a request to the server for synchronizing and uploading its cached data. Once uploaded successfully, it is unpacked and examined by the server for further automated processing.

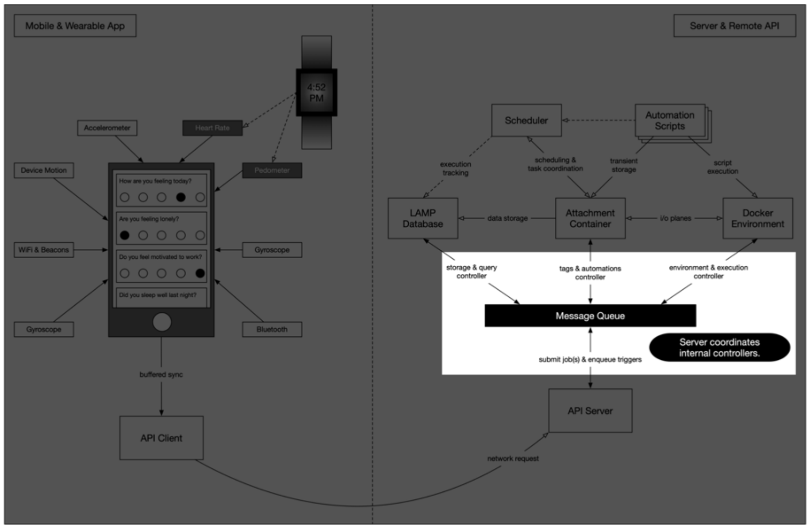

Step 4: Server coordinates internal components for processing.

The server prepares instructions for internal components based on the data uploaded by the app. These instructions are pushed through the internal data bus (a message queue) that connects all internal controllers in the platform. The extensibility of the internal components and the data bus interconnecting them means components can be swapped out or replaced depending on context. In deployments or regions where some features should be disabled, their relevant components are "unplugged" from this data bus.

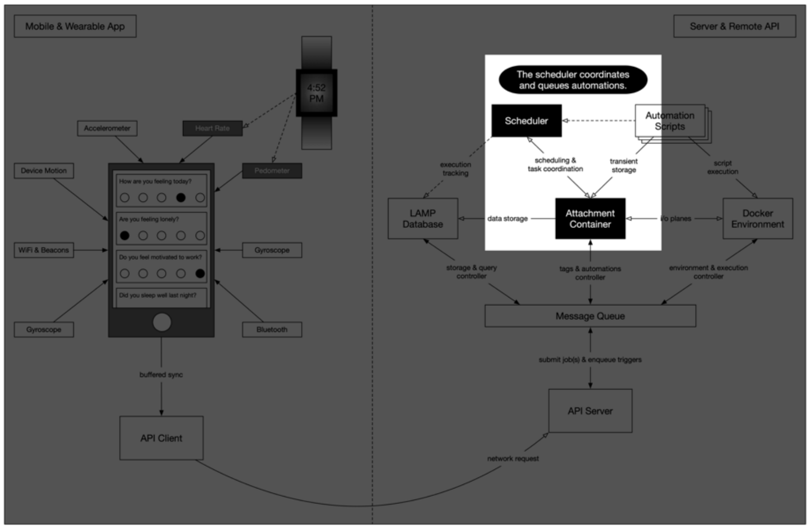

Step 5: Database records the incoming data.

Depending on the server's system configuration and the content of the data uploaded, the database then records all event data and schedules an automated backup. The database considers the origin of the events as it saves them, harmonizing data from various sensors through a unified schema.

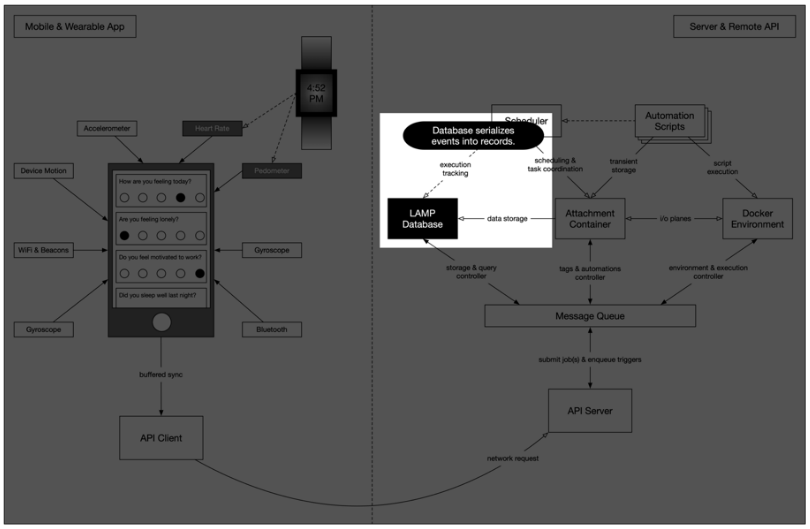

Step 6: The scheduler coordinates and runs automations.

Once the database completes saving data and any backup processes, the scheduler component is engaged to determine if any data processing "applets" or other internal maintenance tasks need to run. After assessing cost and priority, the scheduler creates an execution plan consisting of the above and notifies these components. The scheduler relies on heuristics gathered from audit logs to determine the plan.

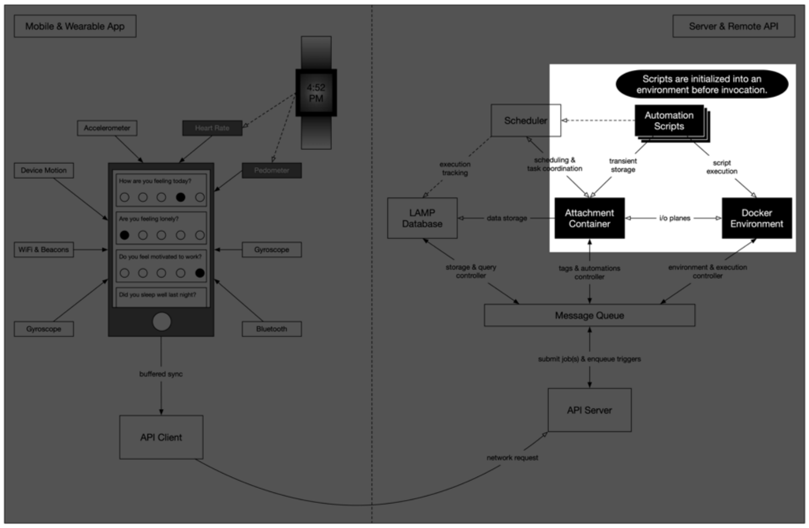

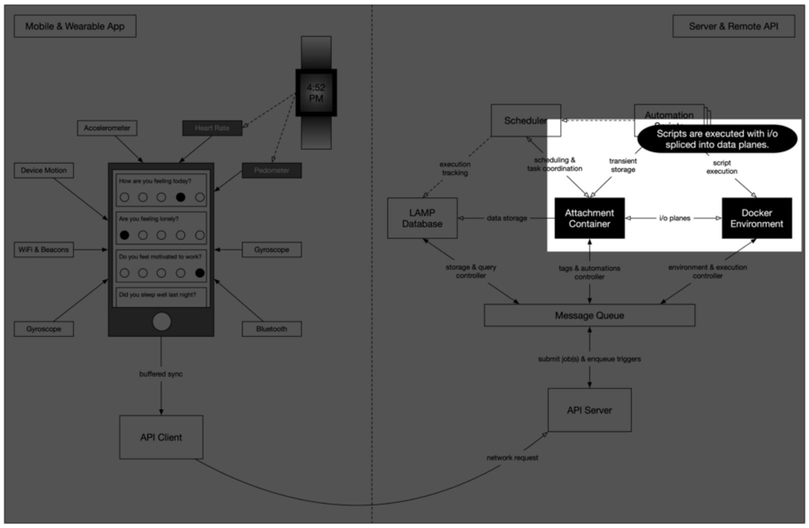

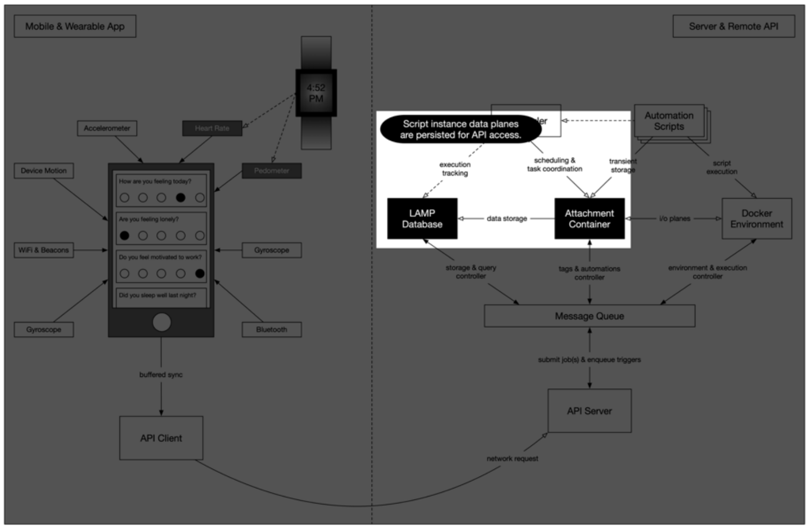

Step 7: Applets are launched into a safe environment and run.

Assuming no internal maintenance tasks need to run, the scheduler may create an execution plan with only a single applet. Once started, a new virtual environment is prepared and securely isolated from other data. For example, an R environment would analyze the script to run and install the correct versions of all required packages, replicating the environment used by the applet's author.

Step 8: Applet results are saved after being run.

As the applet executes, its input and output are spliced from the automation controller and saved as tags. For example, an applet called "com.test.some_script" may create a new tag “com.test.some_script” attached to some resource in the database, or append to an existing tag with the same name. Any runtime logs are extracted separately from this result.

Step 9: Applet results are persisted for later access.

If an applet is configured to persist its output, the data are persisted to the database as a tag and may be accessed by a client app directly at a later time. For example, an applet may compute a dynamic visualization to be cached, or it could lookup login credentials from a predefined tag to access and convert data from a third-party system such as Fitbit into native resource objects which are then persisted by the database.

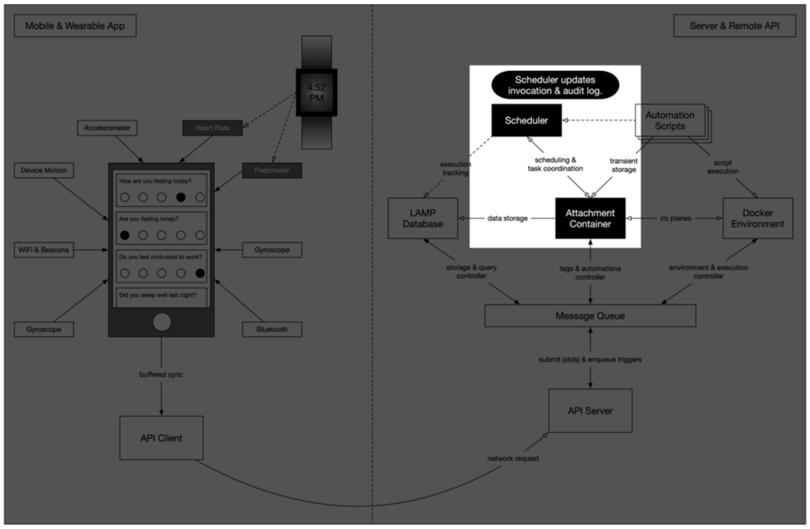

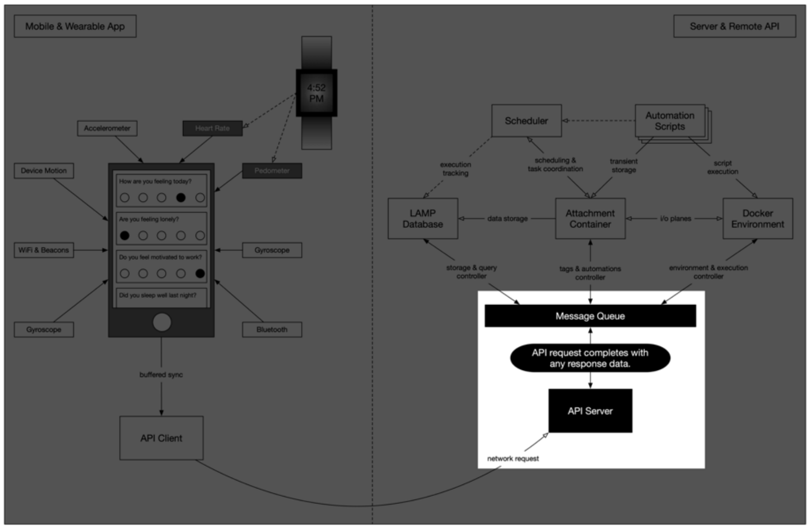

Step 10: Scheduler updates invocation and audit log.

Once an execution plan completes, the scheduler records statistics about it for its next engagement.

Step 11: Request completes with any response data.

If any controller responds to their currently executing instruction with a response payload (such as the execution output of an applet), it is bundled and returned to the API client synchronously. If a controller needs more time to process an instruction, it can return a pointer to an operation resource that can be used in a later asynchronous request to the server to locate the response once completed. If a controller chooses to respond to an instruction but is unable to complete it, the response returns immediately to the client app as an error.

LOW POWER & CONNECTIVITY SUPPORT

The LAMP server encompasses two main purposes:

1. communicate via the LAMP protocol as an API to any participating clients, and
2. store data in the LAMP protocol data format.

As the client of the LAMP server need not worry about the data storage, the semantics of how the data is stored as well as where, or for how long, are transparent and are subject to change in real-time. As long as the client uses the API, all storage access is transparently cached, proxied, or retrieved from a prespecified medium.

We'll use in context the examples of Patient A, in metro Boston with an LTE-connected (high speed cellular data) Apple iPhone 6S+ and an Apple Watch, and Patient B, in rural India with a Xiaomi Redmi GO Android phone and limited spotty cellular data. While both are patients with mental health needs, the differences in their environments and in their devices could impact the quality and availability of the features LAMP is able to provide.

While Patient A sometimes may not be able to charge their devices or access the Internet, Patient B, as well as his or her clinicians, face significantly larger barriers. Patient A is able to access a number of WiFi hotspots and subsidized cellular data, where Patient B may not have access to WiFi at all, with only limited cellular data coverage to rely on.

Suppose a hypothetical situation in which both Patient A and Patient B begin to report increased psychotic symptoms, depressive mood, decreased physical activity, and increased suicidality. A typical app requiring both constant network connection and high frequency sensor data collection to deliver an intervention could respond to Patient A's changes adequately. There is a tremendous chance that it might not, however, respond to Patient B and deliver the correct intervention on time, due to Patient B's environment; even the slight likelihood of such an occurrence would not be considered acceptable if using the LAMP platform.

The proxy mode feature developed core to the LAMP platform allows us to achieve an isolation between factors external to the app, WiFi or cellular data for example, and operating requirements internal to the app. This means that the app will work the same way and deliver the correct interventions on time for both Patient A and Patient B.

In proxy mode, an instance of the LAMP server can continue to vend the API without being attached to permanent/long-term storage. This process requires:

1. a data cache,
2. a connection to another instance of the LAMP server, and
3. periodic synchronization between (1) and (2) determined by an availability factor.

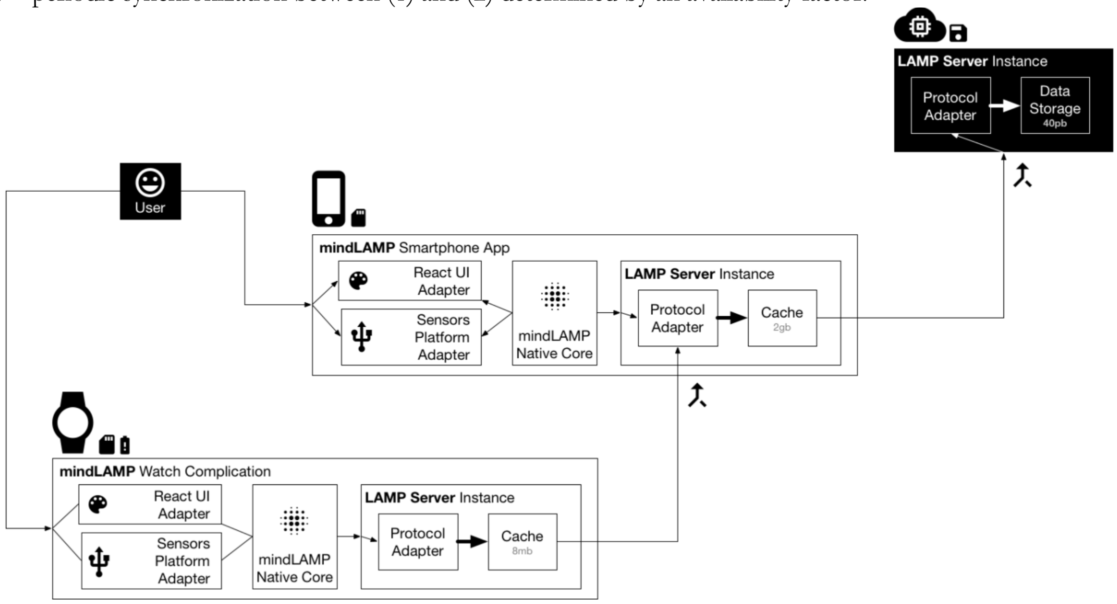

The proxy mode use-case of the LAMP server enables chaining instances together for accumulative data transfer. This serves useful for several reasons:

1. network availability no longer impacts the API client as long as sufficient storage space is available for caching.
2. ActivitySpec updates (that is, when the code underlying a cognitive test or intervention, for example, is updated) are automatically propogated to all instances downstream of the first non-proxy instance when synchronization occurs.
    a. thus, an "application update" not involving the native code of the API client occurs transparently.
3. connectivity method is not specifically defined; though it becomes the concern of the specific instance, it allows for flexibility of transfer between WiFi, bluetooth, LTE, etc. as needed or as capable by the hardware.

As battery and storage size are concerns that impact the overall cost of a smartphone as well as how often it must be charged, Patient B as well as patients with lower economic ability, for example, may not be able to sustain high frequency sensor collection while simultaneously lacking consistent network connectivity.

By both lowering the collection frequency of sensors and running an app-local instance of the LAMP server configured in proxy mode, Patient B will be able to use the same app, automations, and interventions, at a lower but still acceptable fidelity while incurring less battery and storage penalty.

In contrast, even in capable devices and well-equipped environments, recording high frequency sensor data from multiple devices still require coordination. Patient A would be able to configure the smartwatch instance in proxy mode to connect to the smartphone instance, which itself would be configured in proxy mode to connect to an instance of the LAMP server in the cloud. This daisy-chaining of instances allows the smartphone instance of the LAMP server to effectively "see" the data from the smartwatch instance and be able to perform actions in response to it.

The network-visible model of customizable patient interactions in the LAMP Platform is as follows.

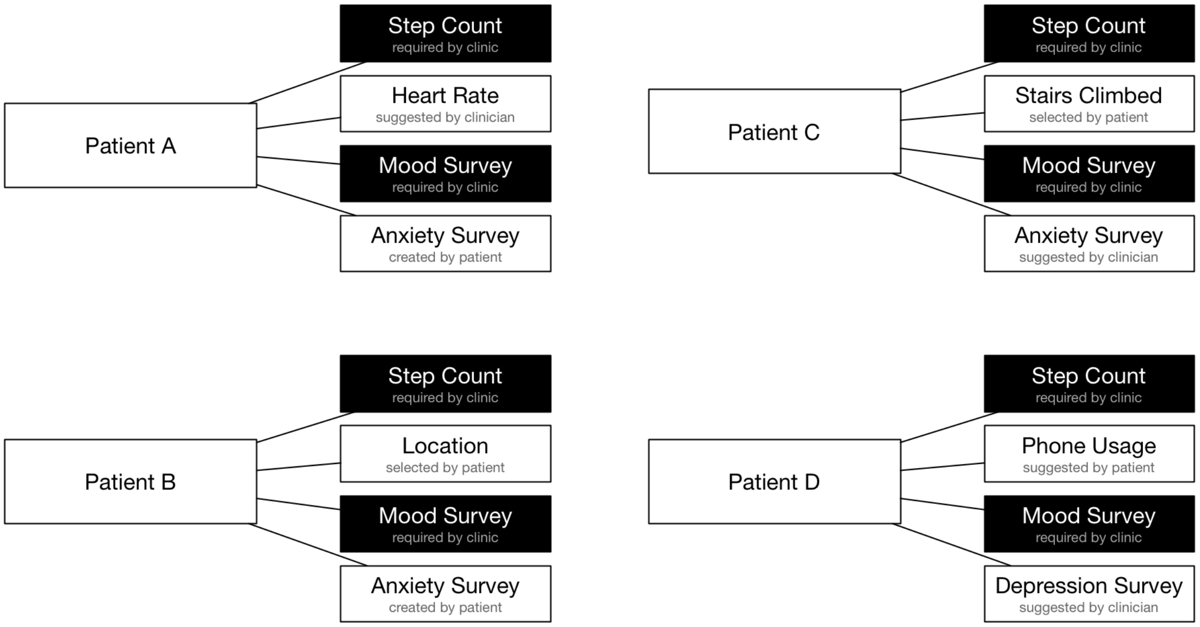

Limitations & Strategies

While a server instance in proxy mode appears transparent to any clients, there are a few concerns followed by mitigations thereof:

1. **origination:** data cached and transferred through several instances in proxy mode would lose meaningful tagging of origin (i.e. from a wearable with high accuracy sensors or a smartphone with low accuracy sensors).
    a. the use of an API key carries origination information encoded as a JWT (JSON Web Token) for all clients irrespective of which server instance they choose to communicate with.
    b. LAMP server instances must only brand their origination upon data if none exists already.
2. **timestamp invalidity:** though the root instance of a LAMP server may be geolocated in the EST (U.S. East) time zone, it may be synchronizing with instances configured in proxy mode geolocated in the IST (Indian) time zone.
    a. LAMP server instances convert timestamps into the GMT (+0) time zone upon receipt from the client.
    b. upon client data access, the LAMP server re-converts timestamps into the local time zone (as specified by an IP address, for example) or the time zone requested by the client.
3. **automation support:** intensive automations such as those written in Python or R cannot be invoked without network availability at the root instance.
    a. some automations, when marked as "lightweight" and written in a supported language, such as Javascript, may be locally cached and invoked on-schedule to facilitate critical and vital functions (i.e. intervention deployment dependent on reported suicidal ideation).
4. **synchronization of non-timestamp-marked data:** update or creation actions on non-event data cannot be synchronized or merged.
    a. such actions can be considered completed by the proxy mode instance but will be queued for synchronization with a timestamp; if the root instance rejects the action, the local proxy mode instance will be reconciled with the most recent data.
5. **Activity deployment-notification and scheduling:** a push notification to deploy an Activity to a patient at the current instance (that is, not a scheduled one) may not succeed if the root instance does not synchronize with the proxy mode instance, and specifically targeting one instance may not be possible (such as the proxy mode instance running on a smartphone instead of on a wearable device, which is unsuitable for interaction).
    a. the API key (that is, the origination as explained above) of a suitable client can optionally be specified when pushing a notification; such notifications will remain queued at the root instance until downstream synchronization reaches the correct proxy mode instance.
    b. if no origination is required, the first available proxy mode instance with the applicable ActivitySpec will consume the notification and dequeue it (preventing downstream instances from knowing it was ever present).

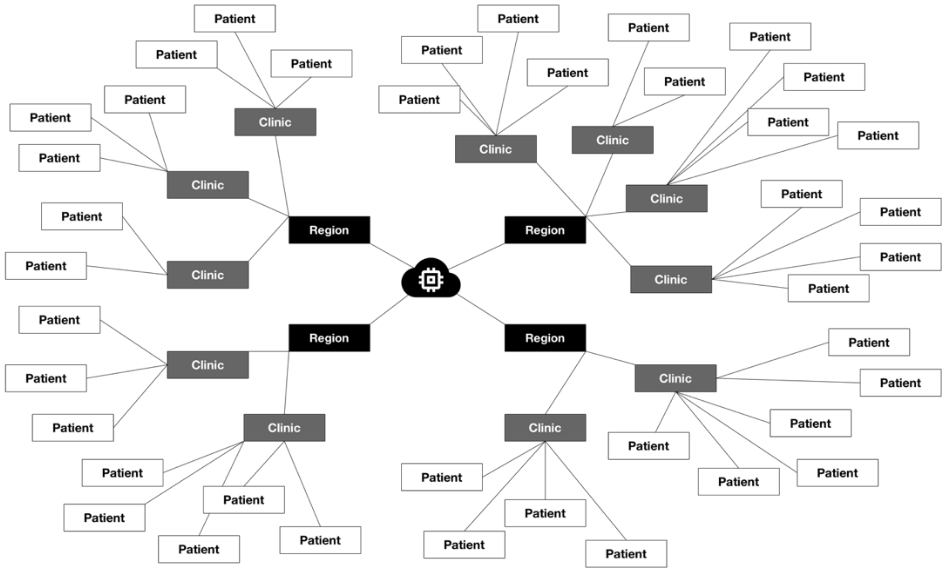

CONTINUOUS MONITORING & INTERVENTION DELIVERY

The primary goal of the LAMP Platform remains the integration of continuous monitoring and rapid intervention delivery. Using the various components of the Platform with the mindLAMP app as an interface could suffice in most situations — processing power and battery life unconstrained, as well as constant access to network resources and the results of the processing from a Cloud server. As a Participant actively enrolled in a study uses the app, including interacting with Activities available therein, sensor instruments periodically collect measurements in the background. These data are submitted to the Cloud server which notifies and activates the correct set of Automations in the right order. These could include Visualizations, Predictive Models, or Clinical Decision Support systems that were installed by the Researcher supervising that Participant's study.

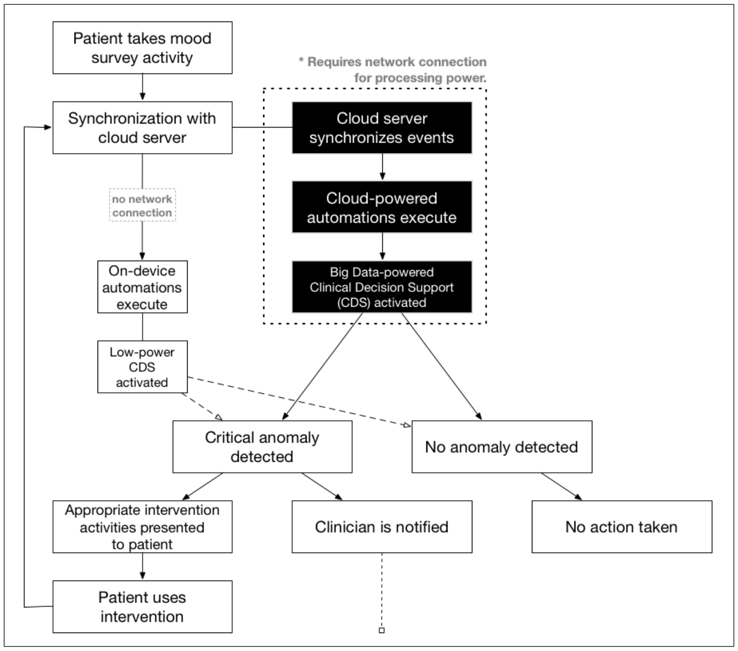

Taking the example of a Big Data-powered Clinical Decision Support (CDS) system, it may ingest a vast amount of passive as well as active data to apply heuristics and derive extracted meta-information, upon which machine learning analysis may detect a critical anomaly requiring clinical intervention and support. After notifying the clinician, this system may notify the Participant with an intervention Activity. As the Participant interacts with the newly suggested intervention, newly recorded measurements from the aforementioned sensor instruments will be synchronized to the Cloud server, and this same CDS system will be invoked again to verify that how successful the feedback was.

Supposing a Participant were not able to remain connected to the network frequently, the mindLAMP app may not be able to communicate with the Cloud server responsible for managing the execution of these Automations. Instead, however, a "low-connectivity" mode is entered, and of all the Automations installed by the Researcher, those specifically designated as capable of low-power processing (and written only in JavaScript, accessing no network or disk resources) will be downloaded and version-synchronized whenever possible. It is this lightweight version of the CDS system that will instead be executed, but the same intervention Activity will be presented to the Participant in the same timely manner. This version of the CDS system might instead rely only on simple heuristic such as the value of a single question related to suicidal ideation, as the processing scale of the Big data-powered variant CDS system would be dramatically greater and unsupportable on smartphone devices.

COMPONENTS

LAMP Protocol

The **LAMP Protocol**, or Application Programming Interface (API), is the formalized inter-component language used by any app or tool connecting to the **LAMP Platform**. It consists of major "surfaces" that describe types of data, actions that may be performed on these types of data, and access and manipulation control of these data. These "surfaces" are designed to be compatible with the Health Level 7 (HL7) organization's Fast Healthcare Interoperability Resources (FHIR) standard resources as well as compliant with the Health Insurance Portability and Accountability Act (HIPAA).

A schema, or data blueprint, is visually and textually presented below for each type of data in relation to other types, along with a description of the properties and actions available. The use of **JSON Schema** at build-time codifies these schema using sets of validation rules and declared links between types and properties. Furthermore, the Spec resource types use JSON Schema at run-time to constrain data from different device sensors or activity interfaces dynamically.

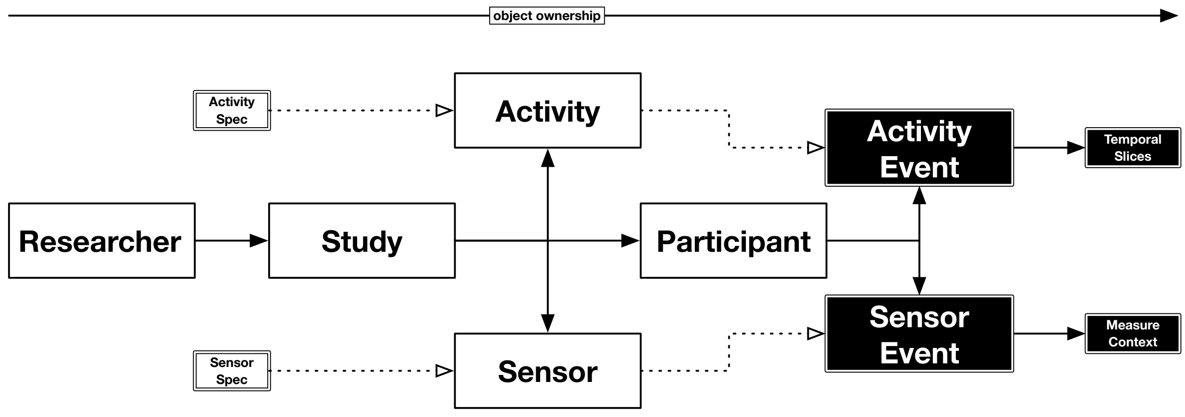

1. **Resource**
    a. **Summary:** an identifier-scoped packet of hierarchically-organized data.
    b. Properties
        i. **id:** the globally-unique identifier for this packet of data.
    c. Actions
        i. **create:** save a new packet of data as a Resource.
        ii. **read:** retrieve the packet of data within the Resource.
        iii. **update:** modify the packet of data within the Resource.
        iv. **delete:** delete an existing Resource.
        v. **list:** retrieve the index of all Resources for a given sub-type below.
2. **Event**
    a. **Summary:** a timestamp-scoped packet of data, part of a time-series data stream ("**event stream**").
    b. Properties
        i. **timestamp:** the millisecond-precision timestamp when this packet of data was recorded.

   c. **Actions**
      i. **append:** save a new packet of data as an Event.
      ii. **remove:** delete an existing Event.
      iii. **search:** retrieve the timestamp-ordered set of Events for a given query.
3. **Researcher**
   a. **Summary:** a container of Studies managed by an administrator.
   b. **Properties**
      i. **name:** the name of the owner of the Researcher resource.
      ii. **email:** the email address of the owner of the Researcher resource.
      iii. **studies:** the set of Studies linked to the Researcher.
   c. **Actions:** see Resource.
4. **Study**
   a. **Summary:** a container assigning sets of Activities and Sensors to a set of Participants.
   b. **Properties**
      i. **name:** the name of the Study.
      ii. **activities:** the set of Activities linked to the Study.
      iii. **sensors:** the set of Sensors linked to the Study.
      iv. **participants:** the set of Participants linked to the Study.
   c. **Actions:** see Resource.
5. **Participant**
   a. **Summary:** a container of streams of ActivityEvents and SensorEvents managed by a user.
   b. **Properties**
      i. **settings:** a group of arbitrary clinical records, including preferred language, set by the Participant.
      ii. **activity_events:** an event stream consisting of active data.
      iii. **sensor_events:** an event stream consisting of passive data.
   c. **Actions:** see Resource.
6. **ActivitySpec**
   a. **Summary:** a specification of a type of interactive activity, such as a game, survey, or intervention tool.
   b. **Properties**
      i. **name:** the user-friendly name of the interactive activity, such as "Jewels."
      ii. **executable:** the bundled HTML/JS code containing the interface to be shown by the app.
      iii. **help:** additional text or media detailing use of the interface.
      iv. **schema:** the data schema of Activity or ActivityEvent objects, in JSONSchema format.
         1. **static_data:** the non-temporal packet of data contained within an ActivityEvent.
         2. **temporal_slices:** slices of interaction contained within a single Event.
         3. **settings:** the collection of possible configuration options for the interface.
   c. **Actions:** see Resource.
7. **Activity**
   a. **Summary:** describing the settings and schedule of an ActivitySpec available to Participants.
   b. **Notes**
      i. Even if a particular ActivitySpec is available, without an Activity resource, a Participant cannot interact with that activity.

ii. Multiple Activities under a single Study may be configured against the same ActivitySpec, such as "Jewels" or "Survey," since each Activity may have different schedules or settings.
1. In the case of "Jewels," an Activity named "Morning Brain Stretch" could be scheduled for MTWTF at 8:00am with moderate or light difficulty; an Activity named "Monday Marathon" could be scheduled for Monday only at 6:00pm with extreme difficulty.
2. In the case of "Survey," an Activity named "Anxiety" would contain questions from the GAD-7 survey instrument; an Activity named "Depression" would contain questions from the PHQ-9 survey instrument.
3. If no schedule is provided, the user will not be notified to interact with the Activity, but it will remain available for the user to willingly use; if no settings are provided, the Activity launches with default values only.

iii. While the ActivitySpec should be considered "static," an Activity is designed to dynamically change if required and manipulated by an administrator.

c. **Properties**
i. **name:** the user-friendly name of the activity, defaulting to the name of the ActivitySpec.
ii. **spec:** the ActivitySpec that constrains this Activity and its ActivityEvents.
iii. **active:** whether this Activity is available or not for the Participants listed.
iv. **schedule:** the user notification schedule, in **cron-compatible syntax**.
v. **settings:** configuration options for this particular Activity.

d. **Actions:** see Resource.

Below: event stream visual representation.

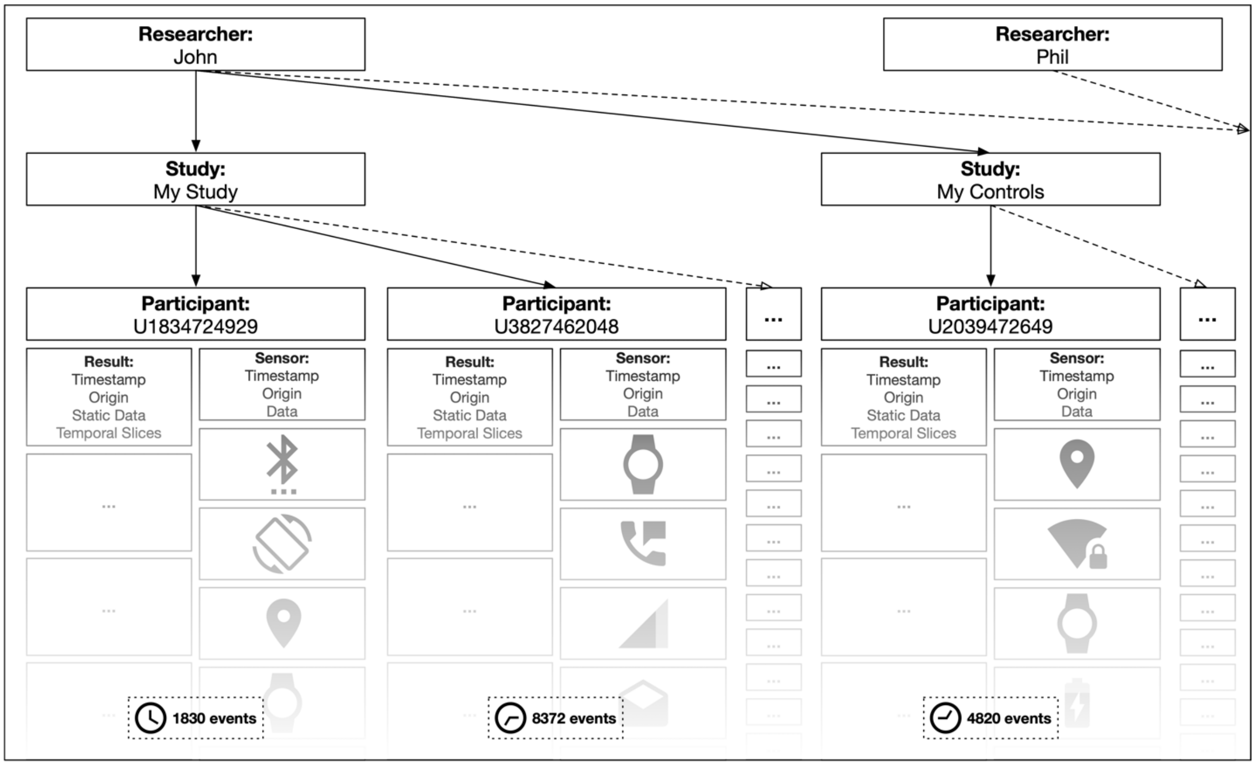

8. **ActivityEvent**
    a. **Summary:** event data stream derived from a Participant interacting with an Activity.
    b. **Properties**
        i. **activity:** the Activity that produced and constrains this ActivityEvent.
        ii. **duration:** the duration of user interaction between recording start and stop.
        iii. **static_data:** the non-temporal packet of keyed data.
        iv. **temporal_slices:** ordered slices of interaction data.
            1. **item:** the item that was interacted with; for example, in a Jewels game, the corresponding alphabet, or in a survey, the question index.
            2. **value:** the value of the item that was interacted with; in a Survey for example, this field would be the question choice index.
            3. **type:** the type of interaction that for this detail; in a Jewels game for example, 'none' if the tapped jewel was incorrect, or 'correct' if it was correct.
            4. **level:** the level; for example, in games with multiple levels, this field might be '2' or '4', but this field is not applicable to surveys.
            5. **duration:** the difference in time from the previous slice.
    c. **Actions:** see Event.
9. **SensorSpec**
    a. **Summary:** a specification of a device sensor available.
    b. **Notes**
        i. It is not possible to bundle dynamically executed code for hardware device sensors.
    c. **Properties**
        i. **name:** the user-friendly name of a device sensor, such as "Accelerometer."
        ii. **schema:** the data schema of Sensor or SensorEvent objects, in JSONSchema format.
            1. **data:** the packet of data contained within a SensorEvent.
            2. **settings:** the collection of possible configuration options for the sensor.
    d. **Actions:** see Resource.
10. **Sensor**
    a. **Summary:** describing the collection frequency and parameters of a device sensor.
    b. **Notes**
        i. Even if a particular SensorSpec is available, without a Sensor resource, a Participant cannot record measurements from that sensor device.
        ii. Multiple Sensors under a single Study may be configured against the same SensorSpec, such as "Location," since each Sensor may have different parameters.
            1. In the case of "Location," an Activity named "Mobility" could collect fuzzed (i.e. anonymized) measurements every second as a facet of physical mobility; an Activity named "SocialGPS" could collect raw measurements hourly as a facet of establishments visited or local weather.
        iii. While the ActivitySpec should be considered "static," an Activity is designed to dynamically change if required and manipulated by an administrator.
    c. **Properties**
        i. **name:** the user-friendly name of the sensor, defaulting to the name of the SensorSpec.
        ii. **spec:** the SensorSpec that constrains this Sensor and its SensorEvents.

iii. **active:** whether this Sensor is available or not for the Participants listed.
iv. **settings:** configuration options for this particular Sensor.

d. **Actions:** see Resource.

11. **SensorEvent**
    a. **Summary:** event data stream derived from a Participant's device sensors passively.
    b. **Properties**
        i. **sensor:** the Sensor that produced and constrains this SensorEvent.
        ii. **data:** the packet of keyed data containing a measurement and associated context.
    c. **Actions:** see Event.
12. **Tag**
    a. **Summary:** a piece of arbitrary data attached to a Researcher, Study, Participant, or Activity.
    b. **Properties:** none.
    c. **Actions:** see Resource.
13. **Automation**
    a. **Summary:** an automatically managed applet that is run in response to event streams or data changes.
    b. **Notes**
        i. The reserved identifier "me" can be used as both the source and destination below.
            1. As the source, the context can be inferred, for example, from the currently authenticated Researcher or Participant.
            2. As the destination, the applet is run upon matching events or changes from only the currently authenticated Researcher or Participant.
        ii. If the destination is not an identifier, but a type of Resource, such as "Participant," changes are monitored across all resources of that type, provided those resources exist within the context of the provided source.
    c. **Properties**
        i. **source:** the owner of the Automation, preserved as the security and execution context.
        ii. **destination:** the recipient object(s) of the Automation, upon which changes are monitored.
        iii. **events:** the Event stream or Resource change notifications that cause applet execution.
        iv. **executable:** the applet code, dependency list, and runtime type: R, Python, JS.
    d. **Actions:** see Resource.
14. **Credential**
    a. **Summary:** access control for a Researcher, Study, or Participant.
    b. **Properties**
        i. **origin:** the resource that defines the scope and ownership of this Credential.
        ii. **description:** a user-friendly description of the credential, such as "API Key" or "Mother."
        iii. **access_key:** either an email address or generated API key.
        iv. **secret_key:** either a salted and hashed password or generated API secret.
    c. **Actions:** see Resource.

Boundaries

When requesting buckets of events (using ActivityEvent::all_by_participant() or SensorEvent::all_by_participant(), for example), you may specify a start and end boundary within which events with matching timestamps are captured. If you specify both start and end such that start == end, you'll restrict the boundary to return only a single event, and if you

don't specify both, you'll un-restrict the boundary and request every event. You can also specify an origin that limits the bucket of events or subscription to its stream to a single type of activity or a single type of sensor. For Activity events (ActivityEvent), you can specify an origin that matches your configured activity or to all activities of a given type. You can request buckets of events from a stream or subscribe to the stream directly and be notified when new events appear or old ones are deleted. This follows a typical WebSocket PubSub pattern.

LAMP Server

The platform server manages internal components and inter-component message-passing. Not all components are intended to be developed in-house, but wherever possible, open source and platform-agnostic projects should be preferred. If a component should be developed in-house, it must be open source. Unless specific requirements preclude its usage, components are to be developed and maintained in the Typescript programming language atop 64-bit Linux. Components should and must be embeddable within Docker containers for both staging and production. No specification or requirement is set forth for orchestration, but Docker Compose and Kubernetes are preferred.

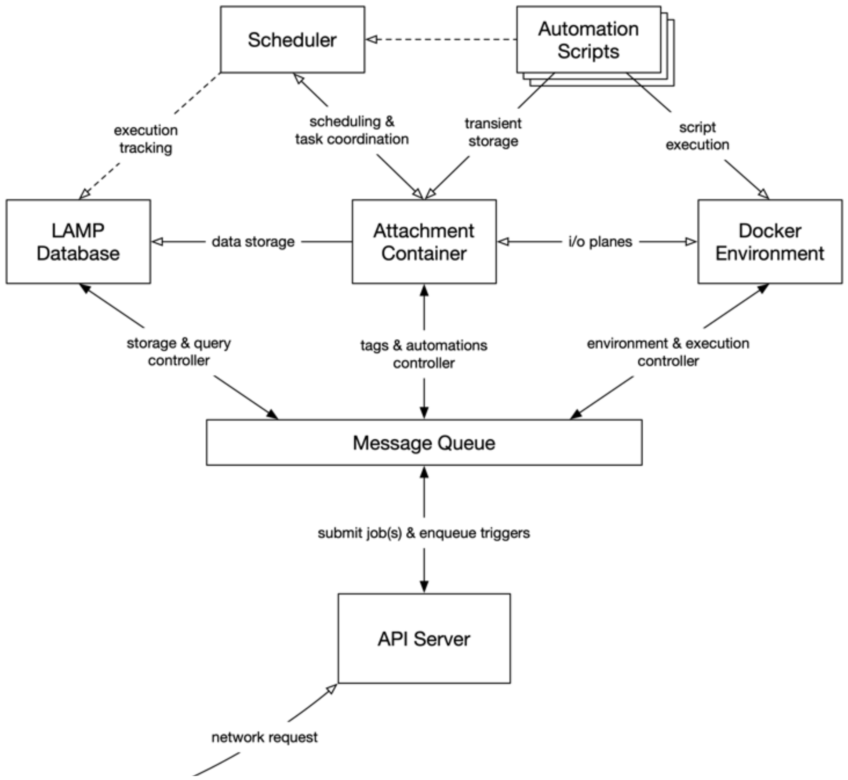

1. **API Server**
    a. **Summary:** the gatekeeper between the internal and external domains.
    b. **Notes**
        i. This component is implemented in **Node.js**.
        ii. All API requests are stateless (i.e. there is no session management) and map to a single response.
        iii. All requests irrespective of authentication requirement must be accompanied by a client API key.
            1. This pre-registered API key is recorded in the audit log with each request and serves as origination reference for resources and events.
        iv. Using the Credential API, requests that require authentication and authorization are validated.
        v. Client app authentication requests are serviced through the integrated **OAuth2** protocol.
        vi. Data transport must be encrypted and decrypted as per security & privacy policy.
2. **Message Queue**
    a. **Summary:** handles the synchronization of communication between all the above actors in the server layer.
    b. **Notes**

i. This component is implemented by the **Redis** high performance key-value store, with an alternate implementation for low-power usage in **Node.js**.
ii. Each component upon startup must register itself with the message queue for service discovery.
iii. Any component may create any number of topics for which other components may publish messages to or subscribe to.
iv. Implementation of database manipulation operations, automation events, and the audit log relies solely on subscription to the global topic; the API Server publishes requests here with a unique identifier awaiting response.

3. **Database**
   a. **Summary:** the persistent data storage device supporting transient caching and complex querying.
   b. **Notes**
      i. This component is implemented by **CouchDB**, with an alternate implementation for low-power usage in **Node.js** using the **PouchDB** framework.
         1. A **legacy adapter** component shall exist to continually await changes (via polling) on the legacy **Microsoft SQL Server** database component and migrate documents to this active database component.
         2. As the **LAMP Protocol** is finalized to support audit log and revisions control, this component shall instead by implemented by **Redis**, **Amazon S3**, or for low-power usage, a naïve **Dictionary/Map** object in **Node.js**.
      ii. A key-value or document database (noSQL) is most ideal for storage needs of the **LAMP Protocol**, due to its hierarchical object data and high throughput access and low latency manipulation requirements.
         1. A relational database (SQL) such as **Postgres** or **SQLite** should serve as an alternate implementation consideration, only as necessary.
      iii. To support real-time intervention deployment and management, an intermediate in-memory cache is used to ease the load on main long-term storage databases.
         1. This portion of the component shall be implemented using **PouchDB** in **Node.js**.
         2. Future implementations should either be **Redis** or **Memcached**.
         3. Data from the cache shall be committed to persistent storage as constraints require.
4. **Intelligent Automations**
   a. **Components:** Docker Environment, Attachment Container, Automation Scripts, Scheduler
   b. **Summary:** text
   c. **Notes**
      i. A compute platform such as a Docker-enabled system or AWS Lambda is used to handle actual execution of code once bundled.
      ii. A dependency bundler such as Webpack, PIP, or Packrat is used to bundle each automation's code to avoid dependency versioning conflicts that could crash or halt execution.

mindLAMP App

The mobile smartphone app is the primary apparatus for the input of Participants' Sensor or Activity events, through interacting with Activities. It consists of several surfaces that work in tandem to convey temporal snapshots of the user to the server. An example of the expected user interface is produced below.

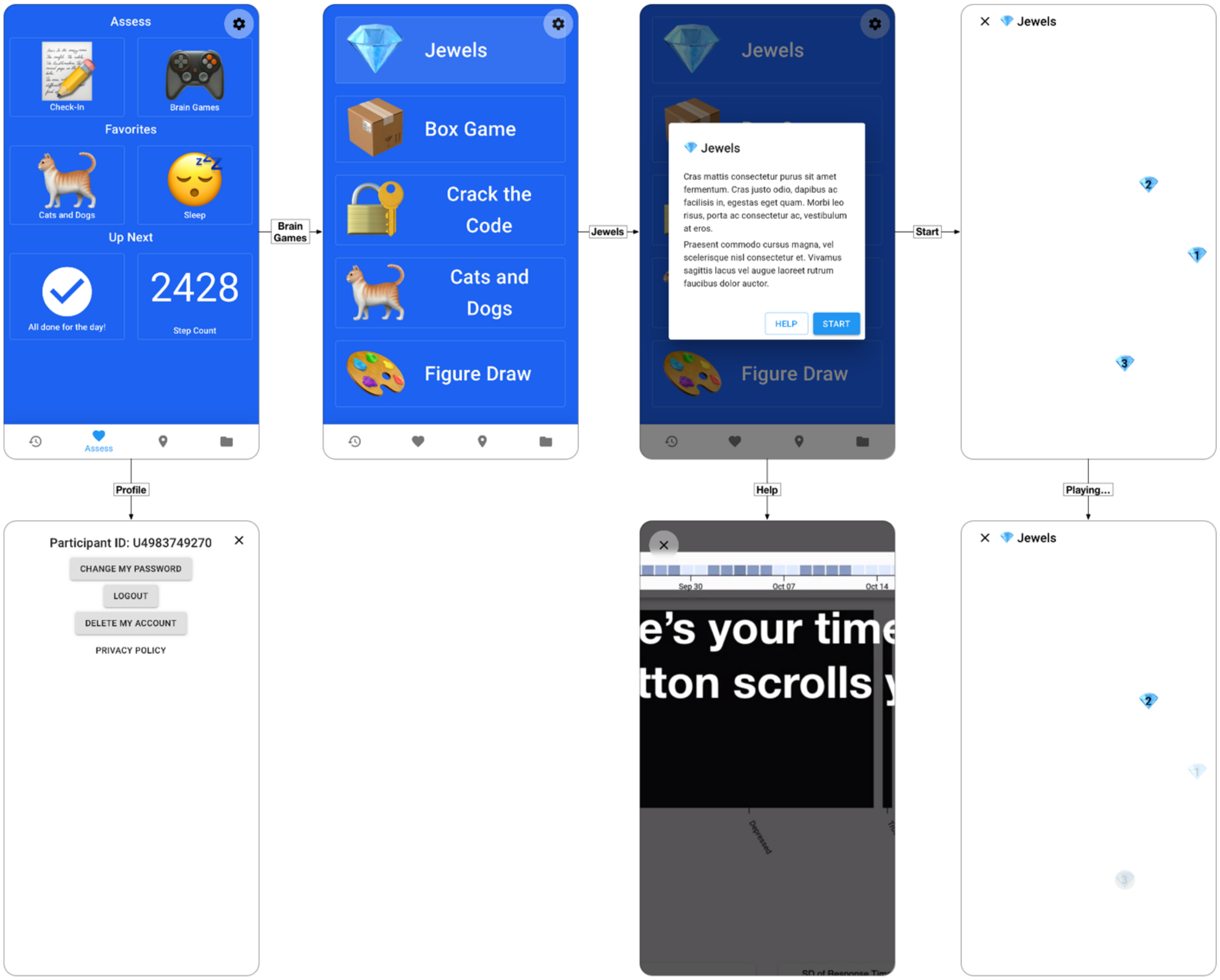

1. **Native Core Surface**
    a. **Sensor Facility:** passive data from sensor instruments is recorded and collected using sensor modules provided by the open source AWARE Framework. Data from Apple HealthKit and Google Fit will be integrated into these data and recorded in tandem.
    b. **Activity View Facility:** source code for Activities as saved in their ActivitySpec definition is downloaded as non-native code and displayed in a Web View.
2. **LAMP API Surface**
    a. **Onboard Server Facility:** to support caching requirements under low power or connectivity modes, an API-compatible but simplified copy of the **Platform Server** can be deployed alongside the native code. (See the "Proxy Mode" section for further details.)

mindLAMP Watch Complication

The wearable complication (app) is the primary apparatus for the input of Participants' Sensor events only, as it remains difficult to design intuitive interfaces for a 1.5" screen.

1. **Native Core Surface**
   a. **Sensor Facility:** we currently plan to record and collect passive data from sensor instruments using the open source AWARE Framework. Data from Apple HealthKit and Google Fit will be integrated into these data and recorded in tandem.
2. **LAMP API Surface**
   a. **Onboard Server Facility:** to support caching requirements under low power or connectivity modes, an API-compatible but simplified copy of the **Platform Server** can be deployed alongside the native code. (See the "Proxy Mode" section for further details.)

mindLAMP Dashboard

The web app serves as a dashboard interface for visualizing, organizing, and controlling all LAMP resources and settings. Visual interface snapshots are produced below, but the most current version of the software is made available at https://dashboard.lamp.digital/ and within the mindLAMP apps, as described above.

1. Authentication Surface
    a. Login/Logout Facility

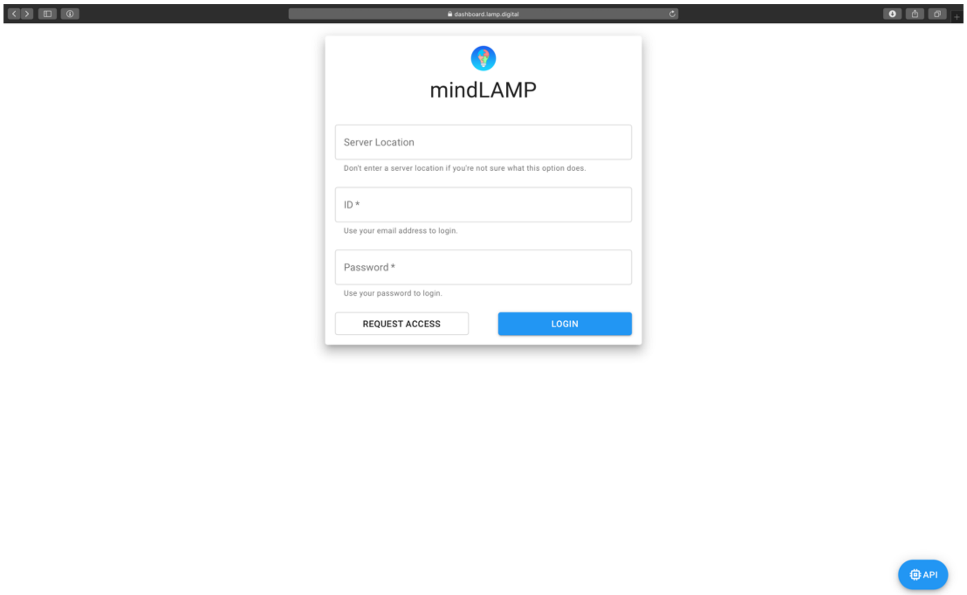

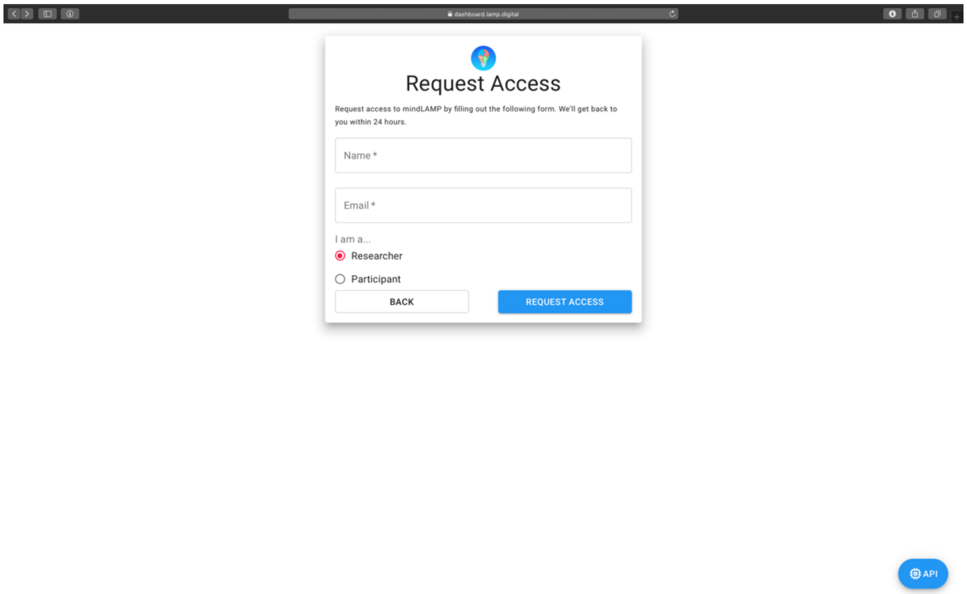
dashboard.lamp.digital
Request Access
Request access to mindLAMP by filling out the following form. We'll get back to you within 24 hours.
Name *
Email *
I am a...
Researcher
Participant
BACK
REQUEST ACCESS
API

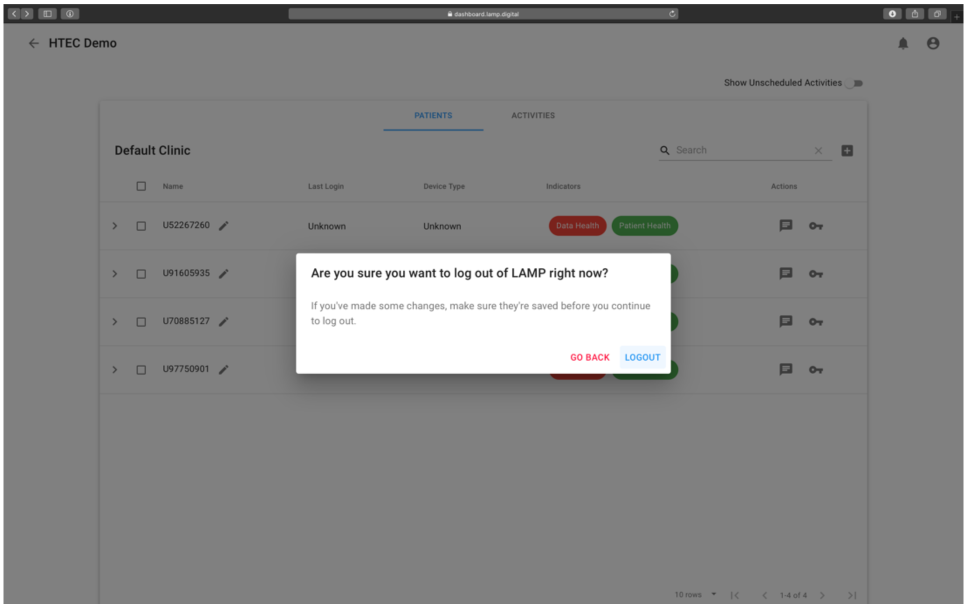
dashboard.lamp.digital
HTEC Demo
Show Unscheduled Activities
PATIENTS
ACTIVITIES
Default Clinic
Search
Name
Last Login
Device Type
Indicators
Actions
U52267260
Unknown
Unknown
Data Health
Patient Health
U91605935
U70885127
U97750901
Are you sure you want to log out of LAMP right now?
If you've made some changes, make sure they're saved before you continue to log out.
GO BACK
LOGOUT
10 rows
1-4 of 4

b. Credential Management Facility

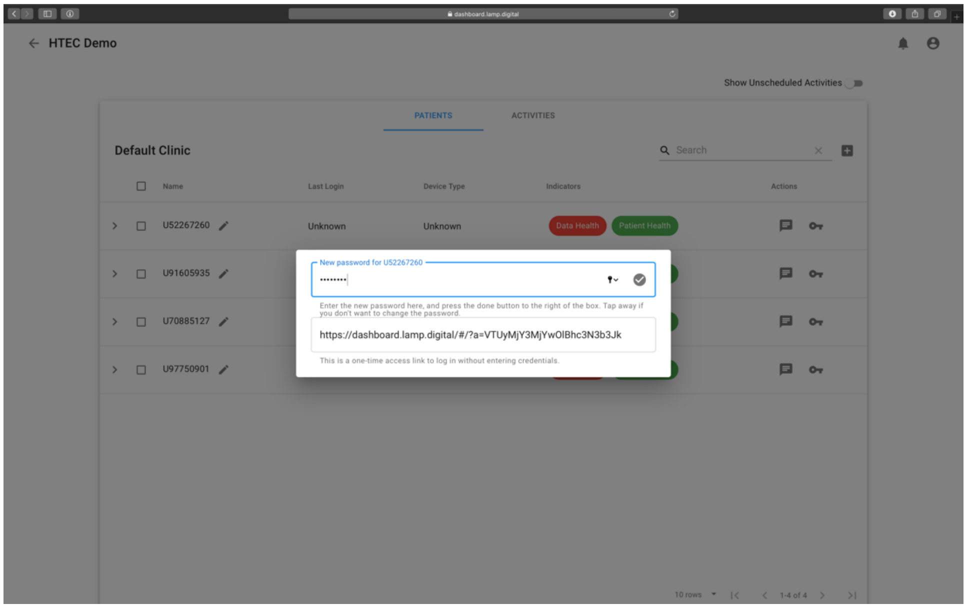

2. System Administration Surface

a. API Control Facility

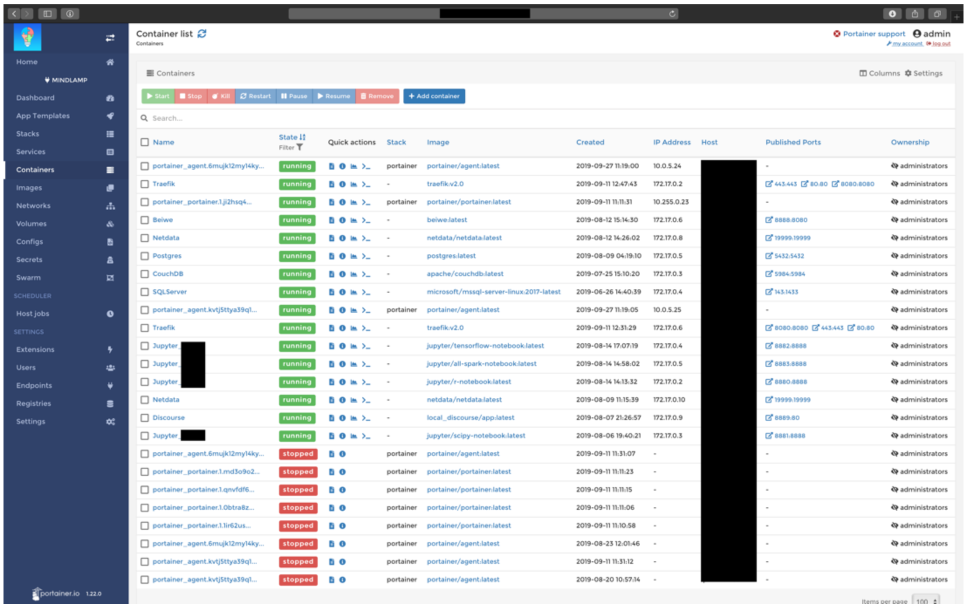

b. API Documentation View Facility

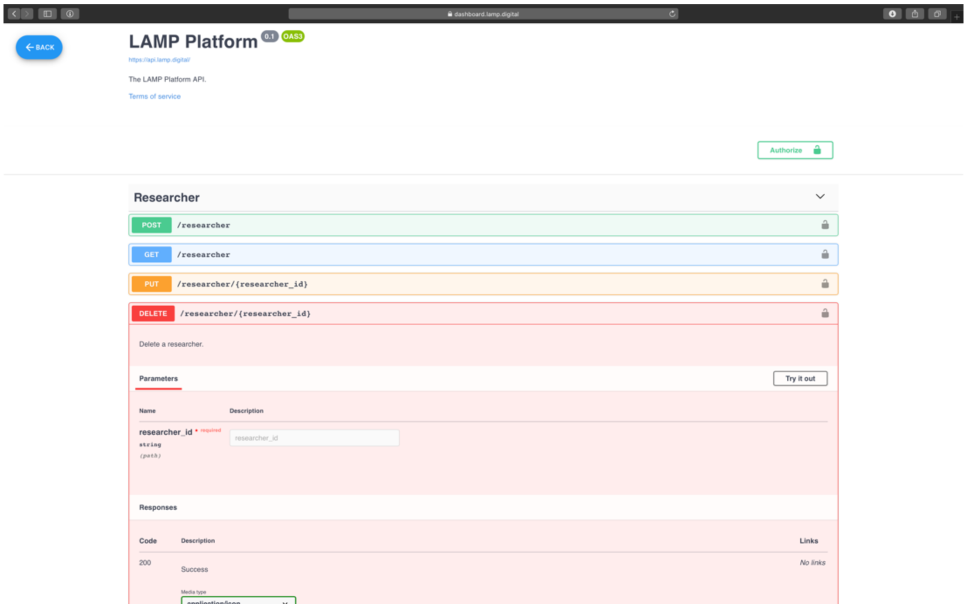

c. Researcher Directory Facility

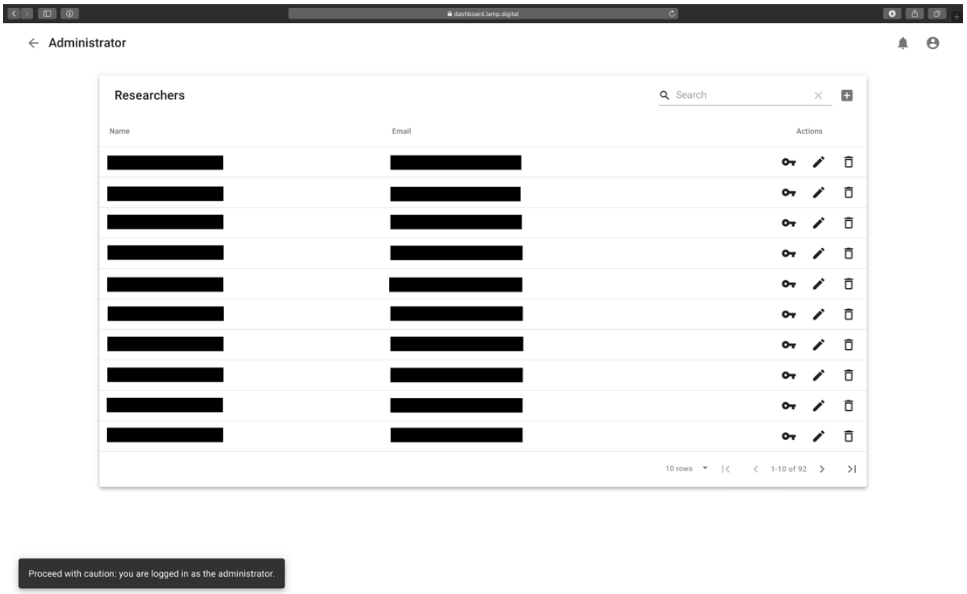

3. Researcher/Study View Surface

a. Summary View Facility

[Undocumented]

b. Participant Directory Facility

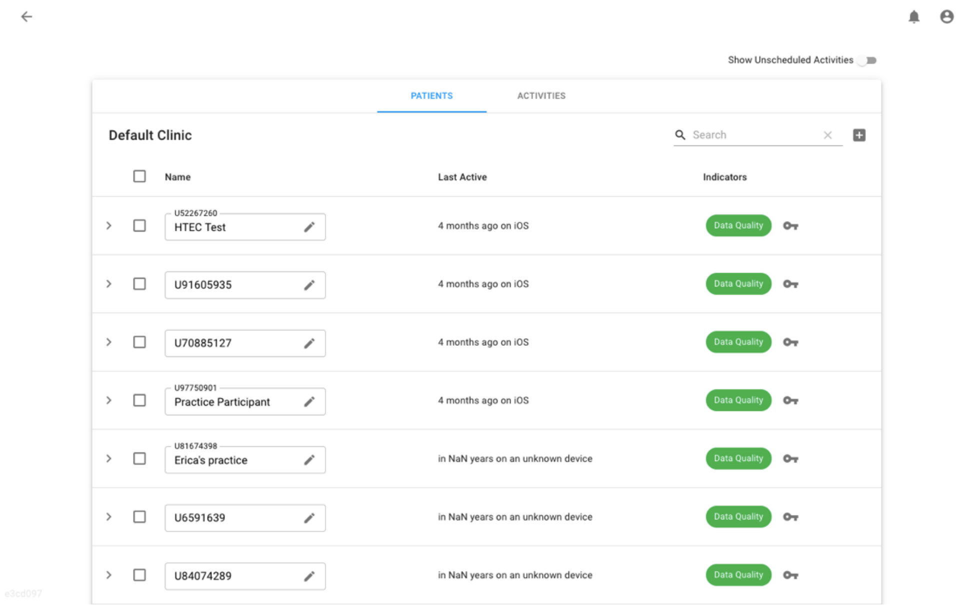

c. Activity Configuration Facility

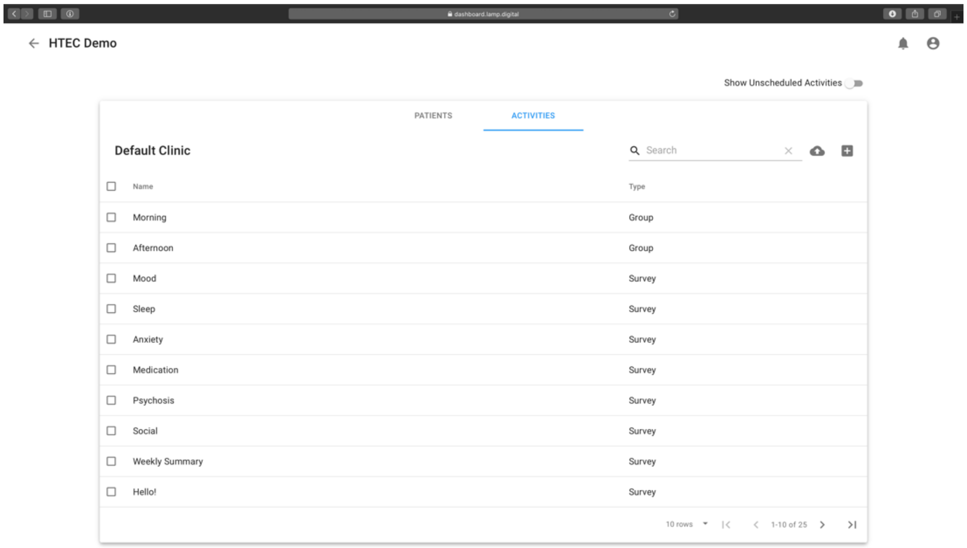

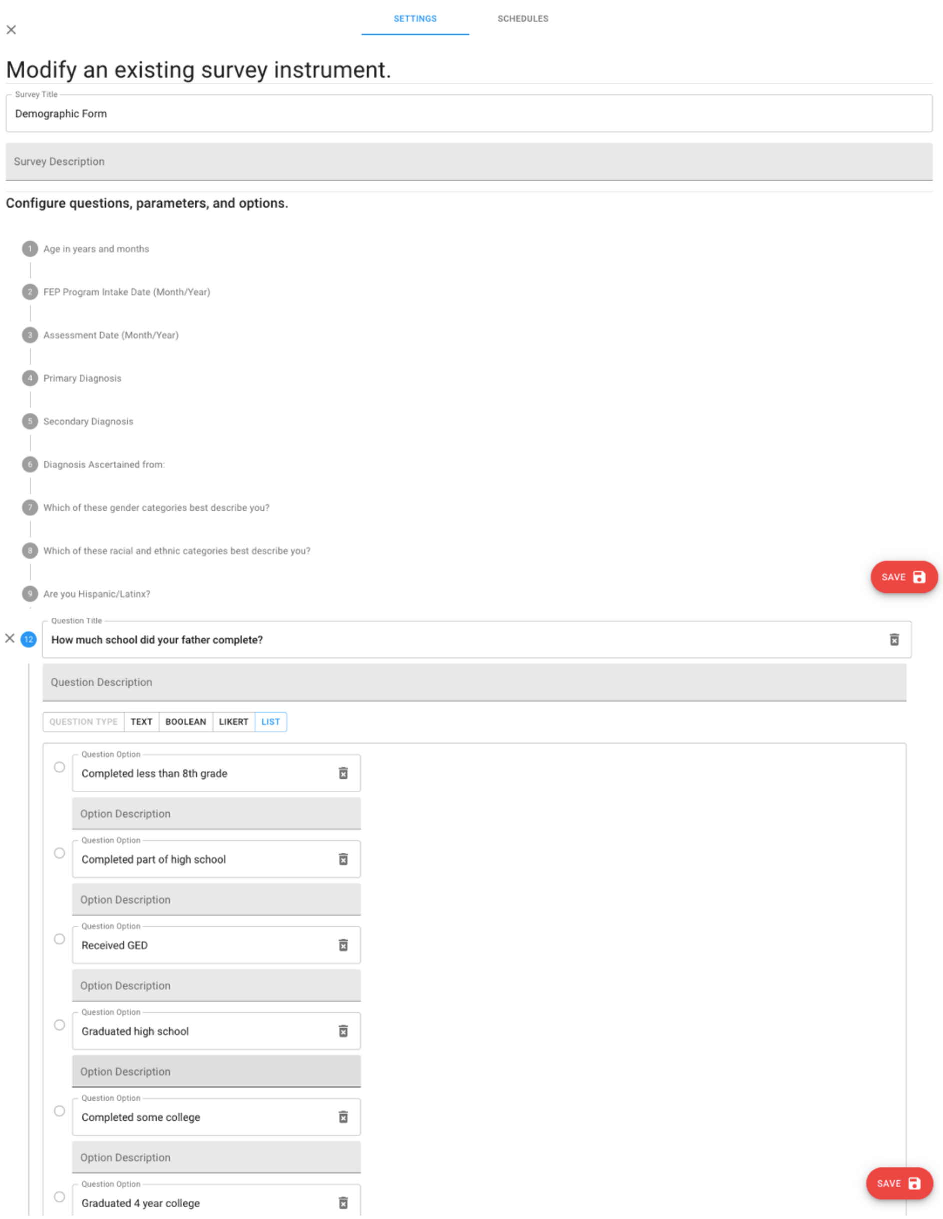

d. Automation/Tag Configuration Facility
[Undocumented]

4. Participant View Surface

a. Visualizations Facility

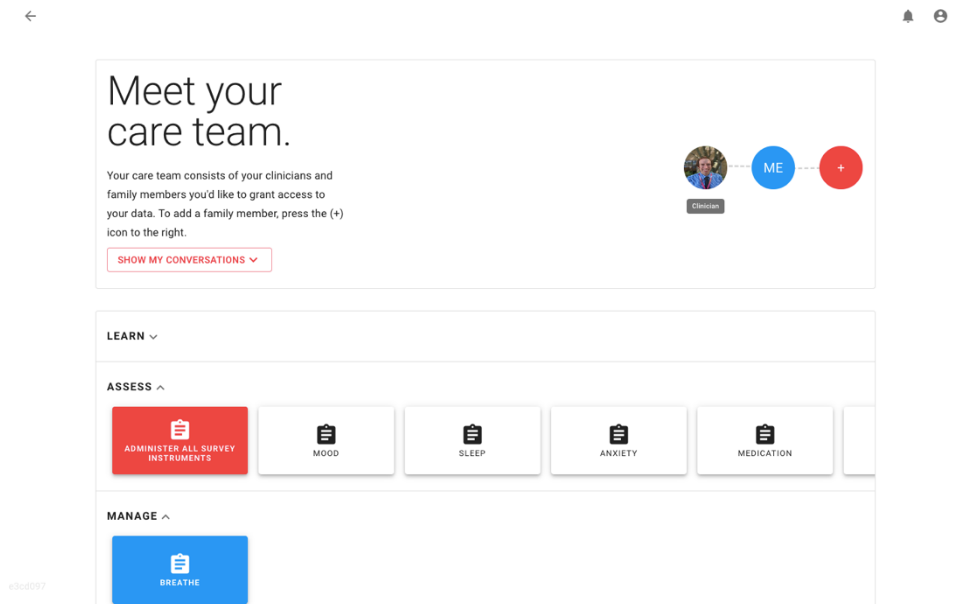

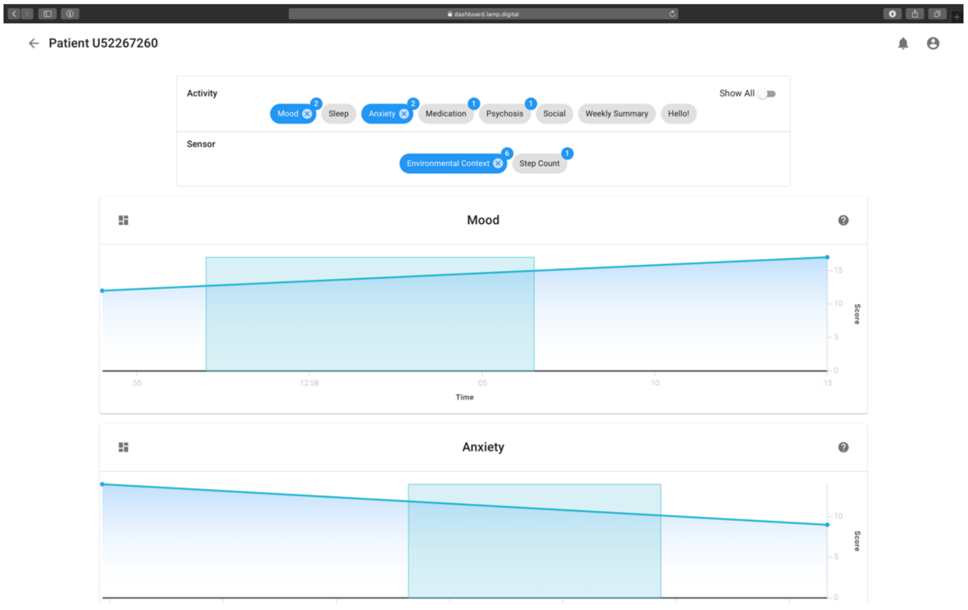

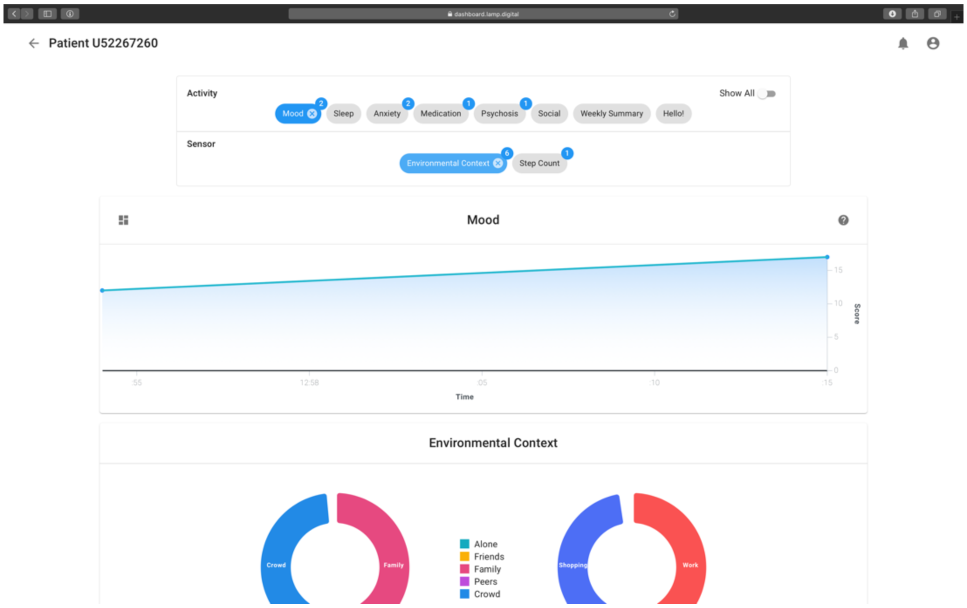
Patient U52267260
Activity
Show All
Mood
Sleep
Anxiety
Medication
Psychosis
Social
Weekly Summary
Hello!
Sensor
Environmental Context
Step Count
Mood
Score
Time
Environmental Context
Crowd
Family
Alone
Friends
Family
Peers
Crowd
Shopping
Work

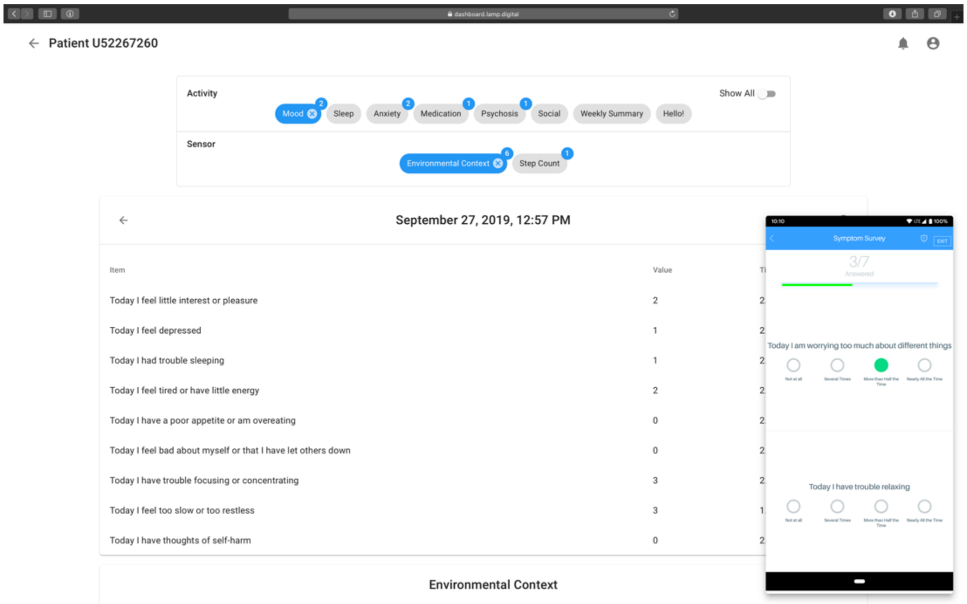
Patient U52267260
Activity
Show All
Mood
Sleep
Anxiety
Medication
Psychosis
Social
Weekly Summary
Hello!
Sensor
Environmental Context
Step Count
September 27, 2019, 12:57 PM
Item
Value
Today I feel little interest or pleasure 2
Today I feel depressed 1
Today I had trouble sleeping 1
Today I feel tired or have little energy 2
Today I have a poor appetite or am overeating 0
Today I feel bad about myself or that I have let others down 0
Today I have trouble focusing or concentrating 3
Today I feel too slow or too restless 3
Today I have thoughts of self-harm 0
Environmental Context
Symptom Survey
3/7
Today I am worrying too much about different things
Today I have trouble relaxing

b. Activity/Survey Usage

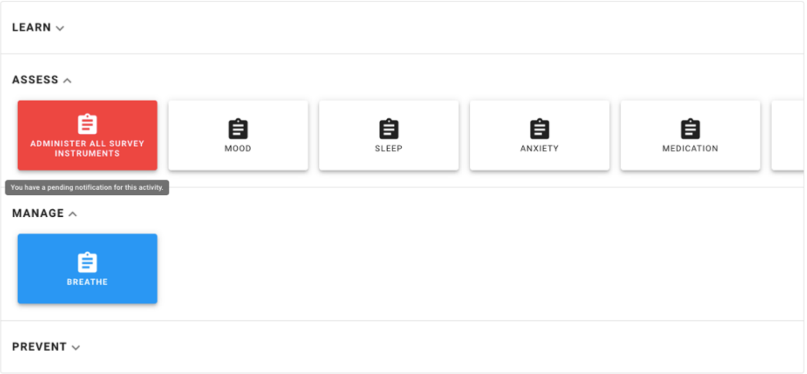

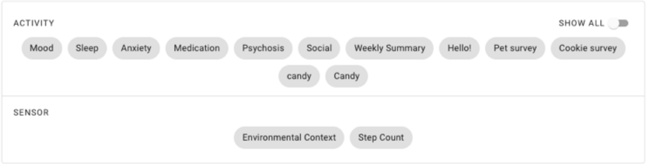

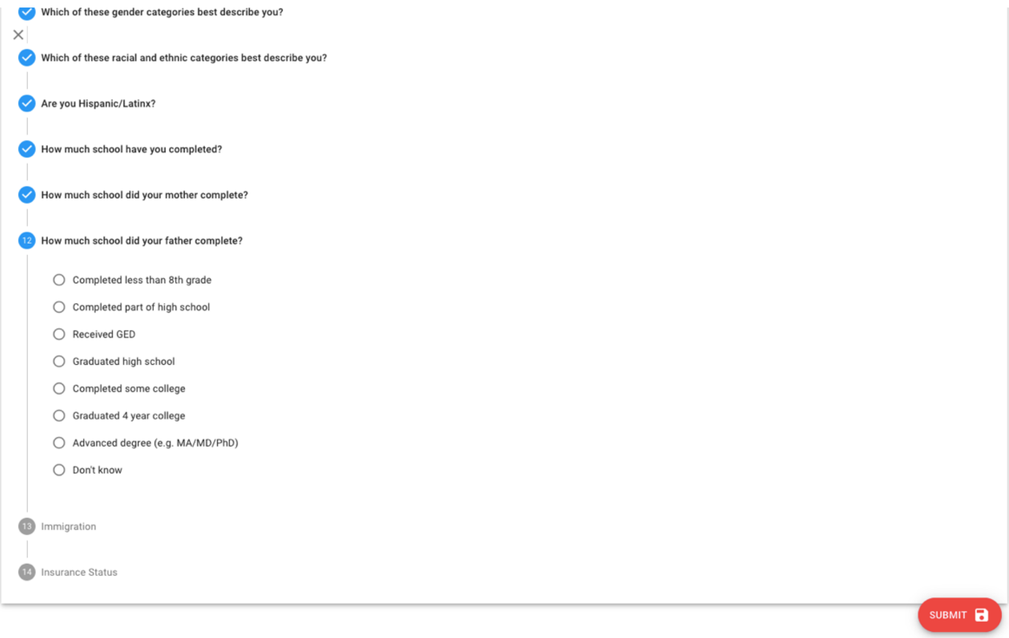

c. Patient Journal/Notes & Clinician Messaging Facility

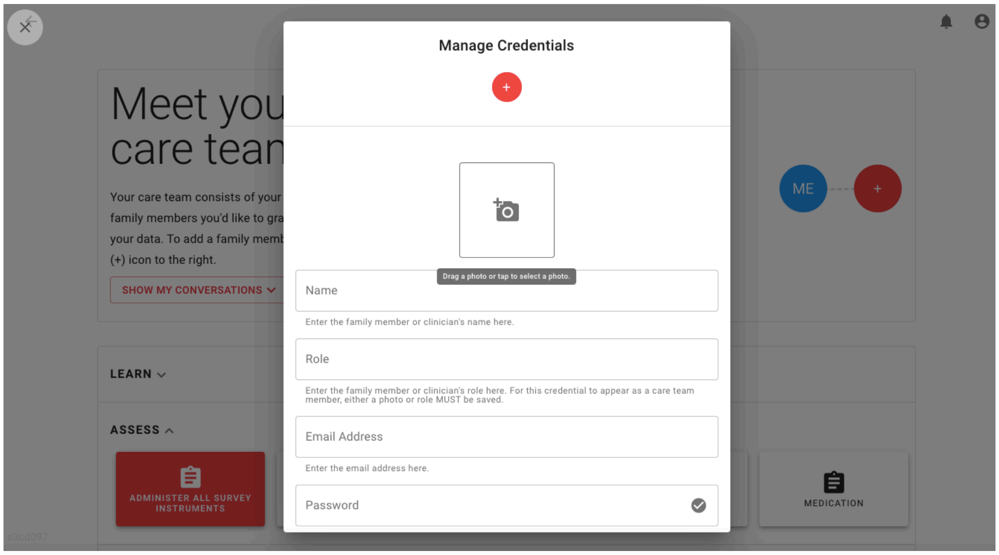

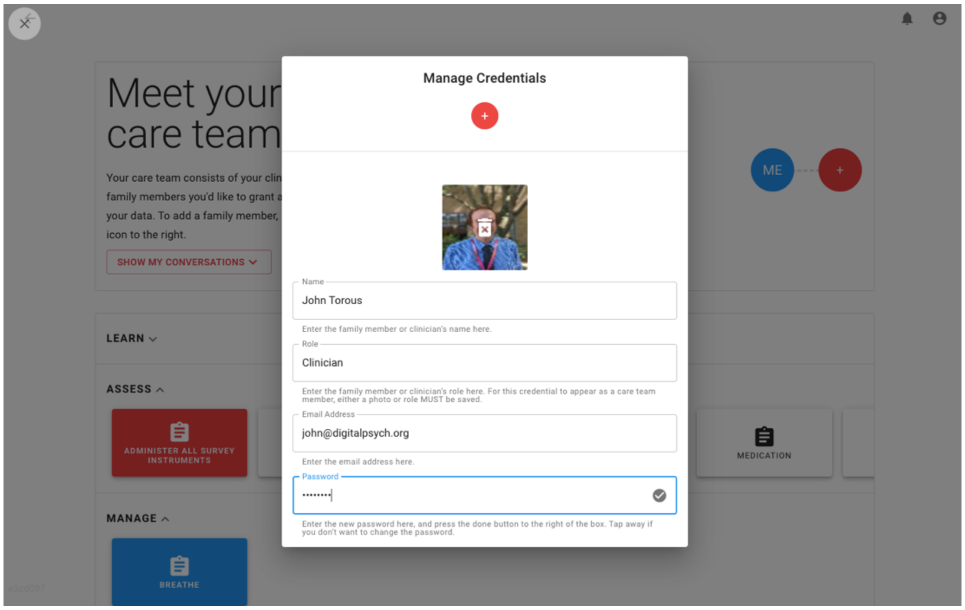

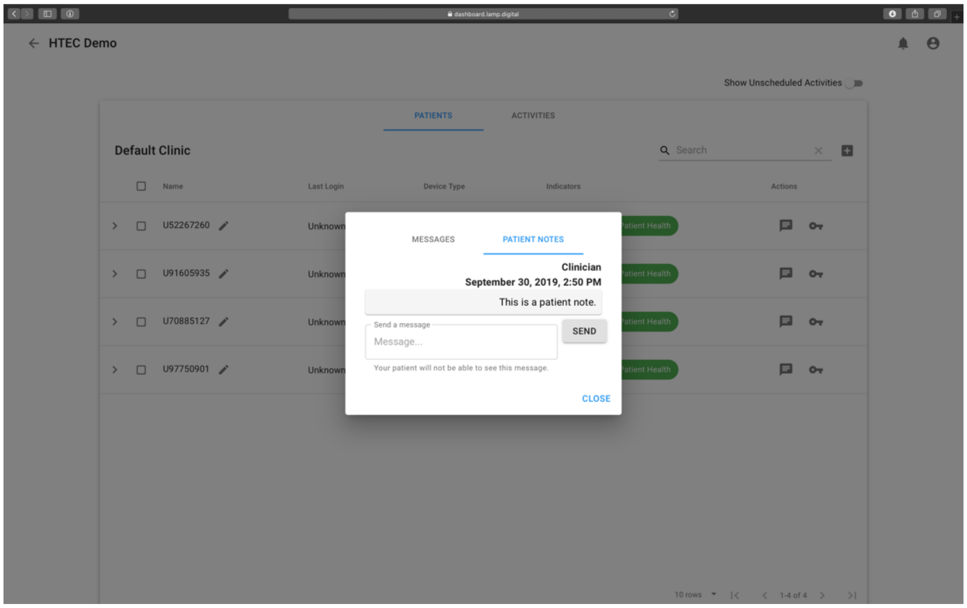
HTEC Demo
Show Unscheduled Activities
PATIENTS
ACTIVITIES
Default Clinic
Search
Name
Last Login
Device Type
Indicators
Actions
U52267260
U91605935
U70885127
U97750901
MESSAGES
PATIENT NOTES
Clinician
September 30, 2019, 2:50 PM
This is a patient note.
Send a message
SEND
Your patient will not be able to see this message.
CLOSE

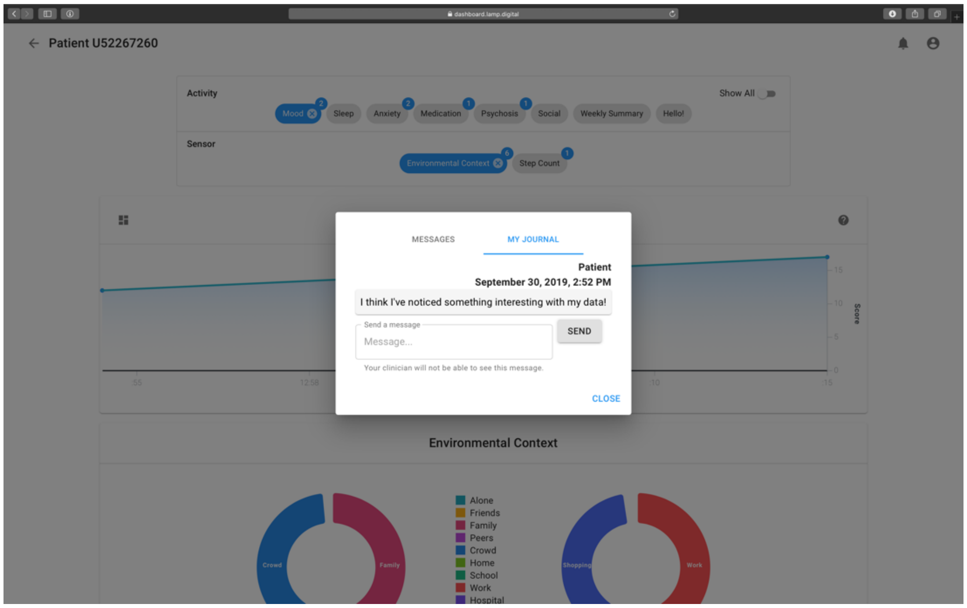
Patient U52267260
Activity
Show All
Mood
Sleep
Anxiety
Medication
Psychosis
Social
Weekly Summary
Hello!
Sensor
Environmental Context
Step Count
MESSAGES
MY JOURNAL
Patient
September 30, 2019, 2:52 PM
I think I've noticed something interesting with my data!
Send a message
SEND
Your clinician will not be able to see this message.
CLOSE
Environmental Context
Alone
Friends
Family
Peers
Crowd
Home
School
Work
Hospital

d. Breathe exercise sample (Activity UI)

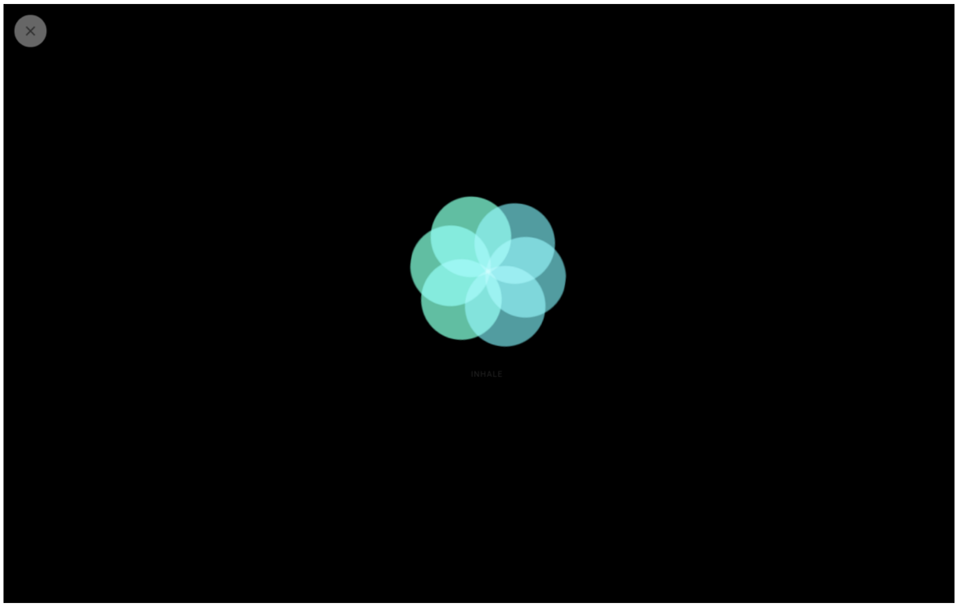

Activities

Active events are produced on a rolling basis via interactions by a Participant. They are transferred to the **Platform Server** automatically by using the Activity API written in JavaScript. By "beginning" and "ending" a recording of these interactions, as well as "emitting" temporal data during the interaction, an ActivityEvent can be captured and sent to the **Platform Server**. Screenshots of currently available Activities are provided below. A live server must be consulted for further information (see GET /activity_spec) and visual or interactive samples are provided in the source GitHub repository.

<table>
<tr><th>lamp.survey</th><th>lamp.spatial_span</th><th>lamp.simple_memory</th></tr>
<tr><td></td><td>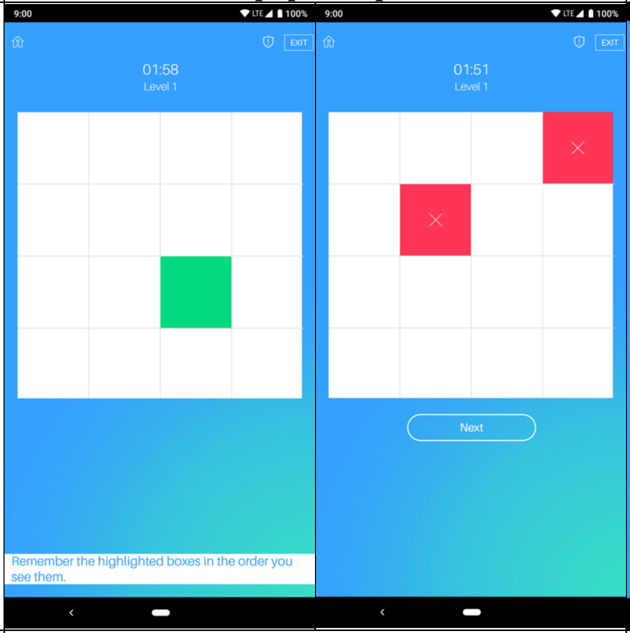
</td><td>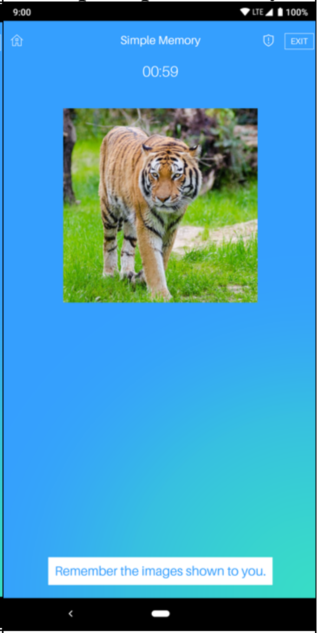
</td></tr>
<tr><th>lamp.serial_7s</th><th>lamp.cats_and_dogs</th><th>lamp.visual_association</th></tr>
<tr><td>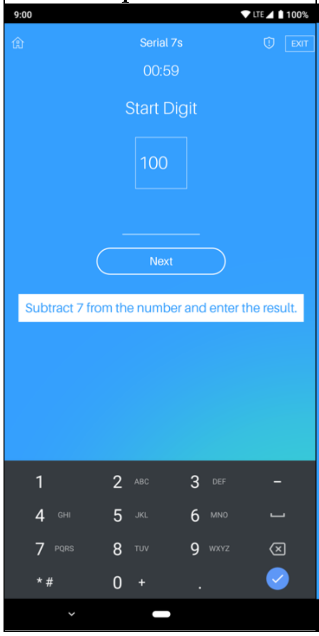
</td><td>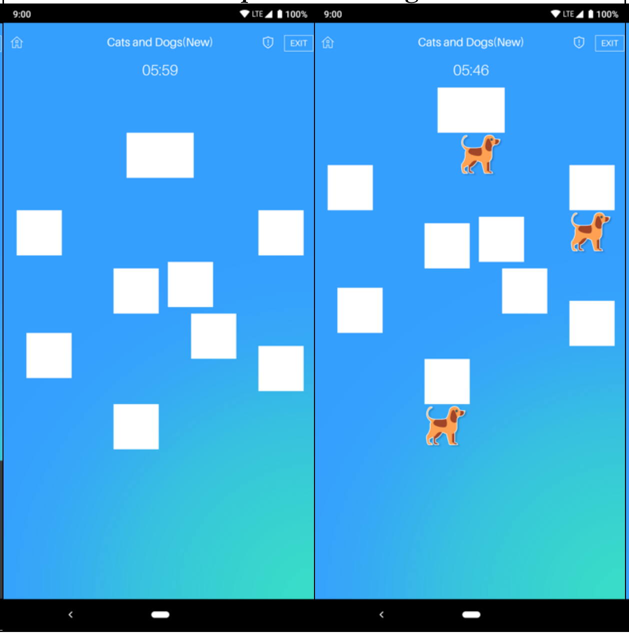
</td><td>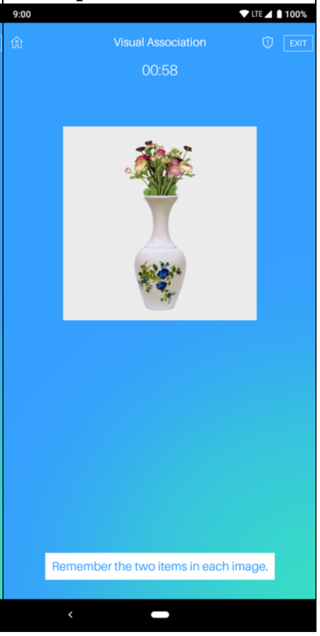
</td></tr>
</table>

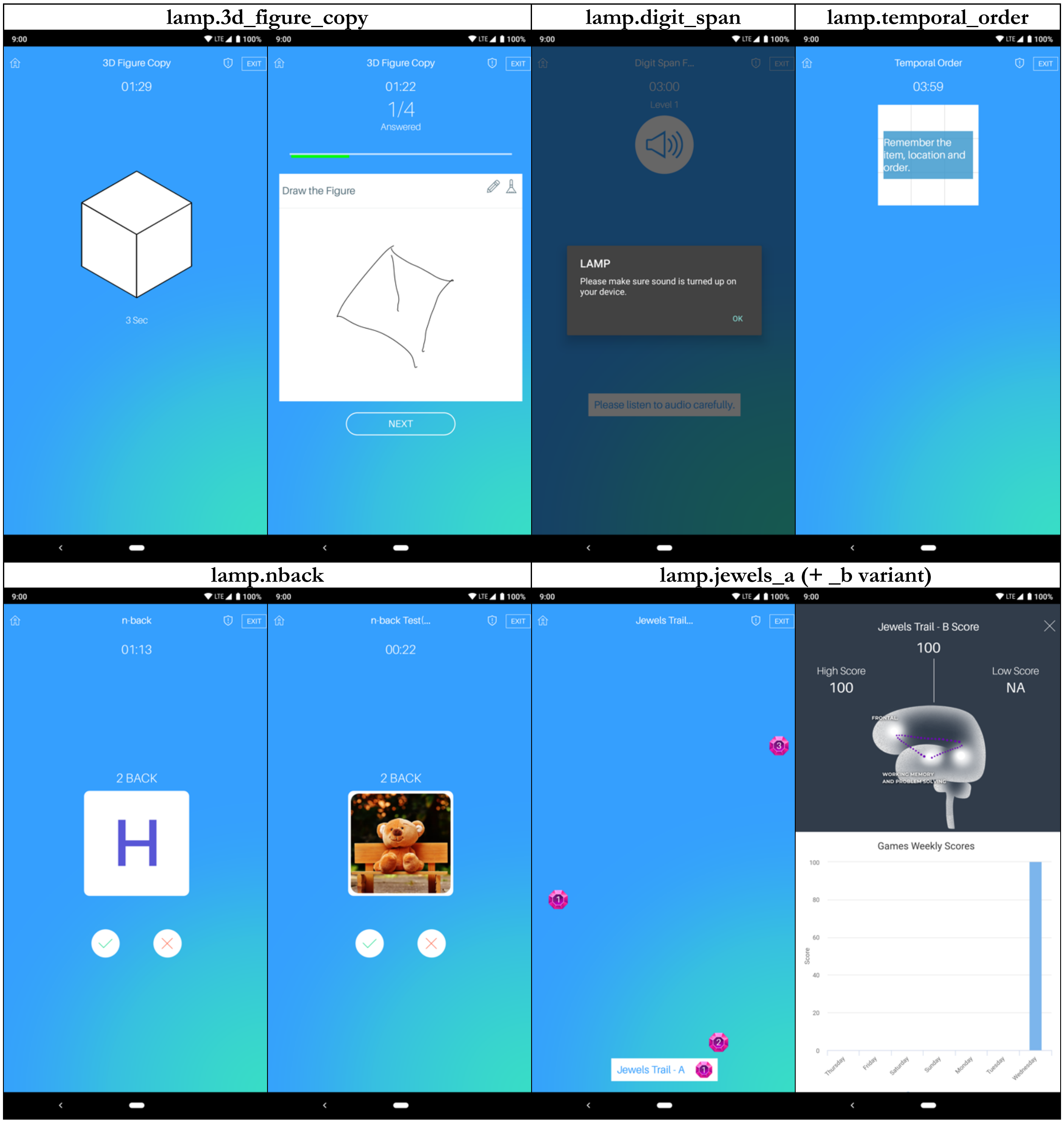
lamp.3d_figure_copy
lamp.digit_span
lamp.temporal_order
3D Figure Copy
01:29
3 Sec
3D Figure Copy
01:22
1/4
Answered
Draw the Figure
NEXT
LAMP
Please make sure sound is turned up on your device.
OK
Please listen to audio carefully.
Temporal Order
03:59
Remember the item, location and order.
lamp.nback
lamp.jewels_a (+ _b variant)
n-back
01:13
2 BACK
H
n-back Test(...
00:22
2 BACK
Jewels Trail...
Jewels Trail - A
Jewels Trail - B Score
100
High Score
100
Low Score
NA
Games Weekly Scores

Sensors

Active sensor events are produced on a rolling basis via interactions by a Participant. They are transferred to the **Platform Server** automatically by using the Activity API written in JavaScript. By "beginning" and "ending" a recording of these interactions, as well as "emitting" temporal data during the interaction, an ActivityEvent can be captured and sent to the **Platform Server**. A list of existing Sensors is provided below with name and description; a live server instance must be consulted for data schema information (see GET /sensor_spec). Implementations for these hardware sensors are provided in the GitHub repository.

1. **lamp.accelerometer:** records unprocessed triaxial accelerometer data.
2. **lamp.accelerometer.motion:** records processed triaxial motion, triaxial rotation, triaxial gravity, and triaxial magnetic field data.
3. **lamp.analytics:** records events such as page opens, notification receipt, or login sessions.
4. **lamp.blood_pressure:** records blood pressure from an external connected monitor.
5. **lamp.calls:** records calls after encrypting the phone number.
6. **lamp.distance:** records total distance moved.
7. **lamp.bluetooth:** records bluetooth devices within range as well as signal strength.
8. **lamp.flights:** records stairs of flights climbed.
9. **lamp.gps.contextual:** records two dimensional GPS location along with user-reported context.
10. **lamp.gps:** records three dimensional GPS location.
11. **lamp.height:** records self-reported height.
12. **lamp.magnetometer:** records triaxial magnetic field changes.
13. **lamp.respiratory_rate:** records calls after encrypting the phone number.
14. **lamp.heart_rate:** records heart rate from an external connected monitor.
15. **lamp.screen_state:** records device usage changes such as lock state and screen visibility.
16. **lamp.segment:** records workout segment duration and length.
17. **lamp.gyroscope:** records unprocessed triaxial gyroscope data.
18. **lamp.sms:** records text messages after encrypting the phone number.
19. **lamp.sleep:** records sleep duration with start and stop times.
20. **lamp.weight:** records self-reported weight, or weight from an external connected monitor.
21. **lamp.steps:** records number of steps taken since last such event, or the start of the day.
22. **lamp.wifi:** records encrypted wireless hotspots as well as signal strength.

CLOUD DEPLOYMENT & INFRASTRUCTURE

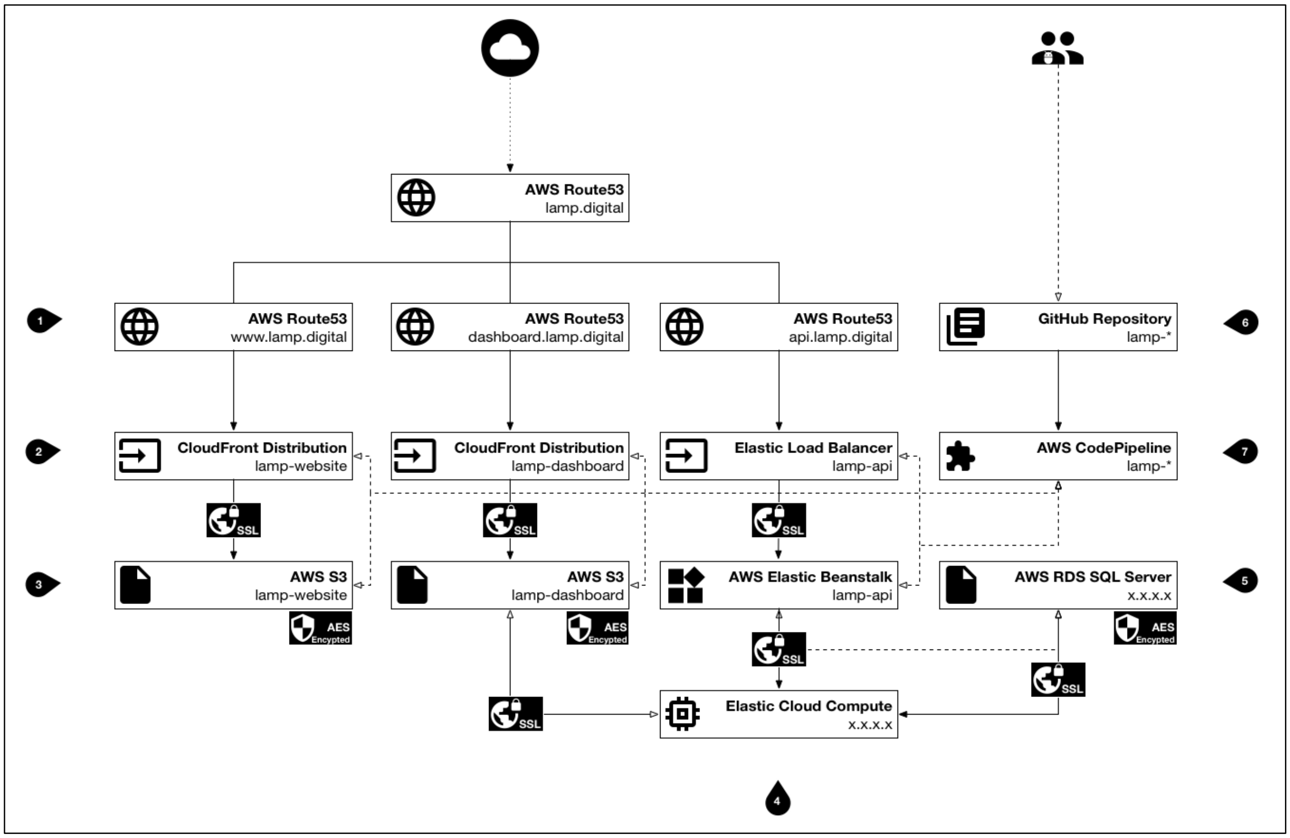

Provisioning Cloud Resources

Though AWS Route 53 is listed in the diagram above detailing a sample architectural plan to host the LAMP Platform, there is no requirement in place for a particular provider. Suggestions made in the diagram above include AWS Elastic Beanstalk, AWS RDS, AWS S3, and AWS EC2; these managed services provide robust and maintenance-free bring-up for the LAMP Platform. The equivalent products in the Microsoft Azure or Google Cloud suite would also be acceptable. Provided with the LAMP Platform is a set of Docker Stack files meant to be used with Docker Swarm (for cloud testing, integration, and production usage) or Docker Compose (for local testing). These files are easily adapted to Kubernetes as well, and all services hosted within the Stack files provided are of an open source nature, with the exception of the Microsoft SQL Server for legacy usage. Please ensure that when deploying the LAMP Platform, all proprietary software is licensed for your use case before deployment.

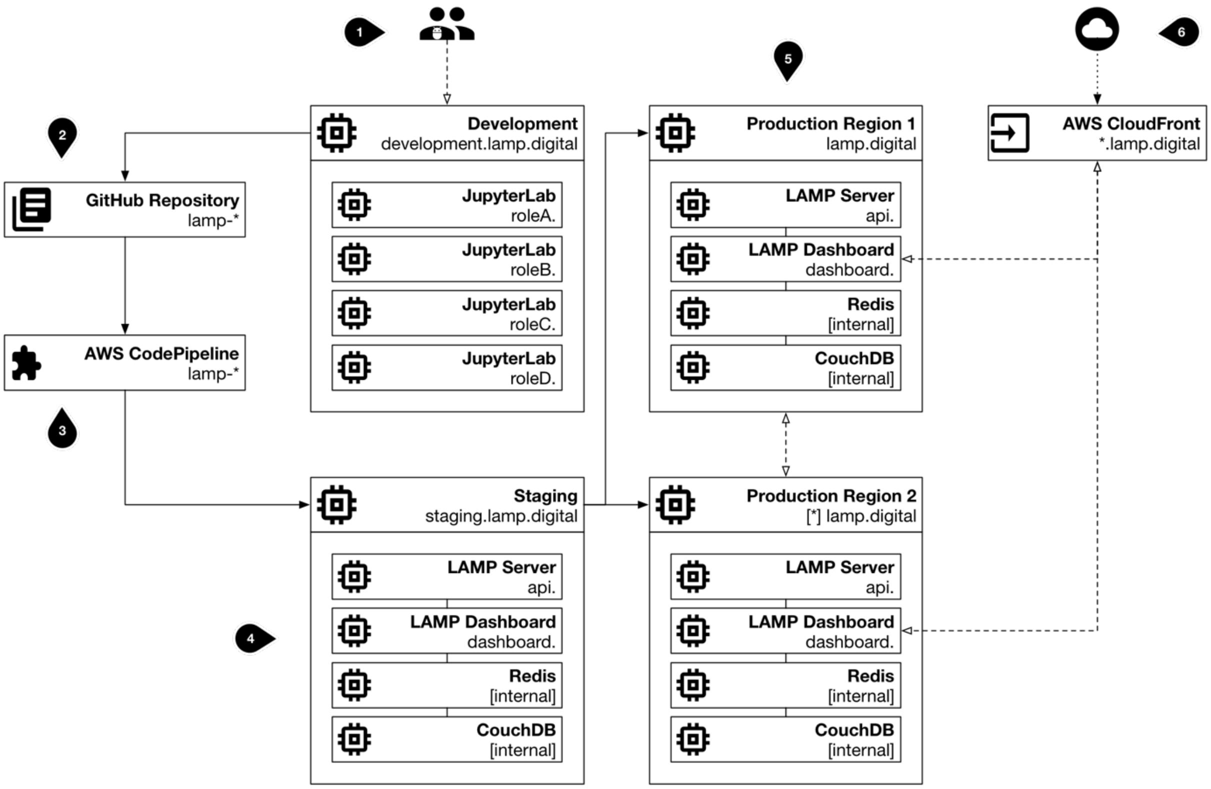

Segmenting Compute Resources

The diagram above describes the GitHub workflow in one possible pathway using AWS CodePipeline to quickly perform continuous testing, integration, and deployment. AWS CloudFront is suggested as a caching content delivery service though others such as CloudFlare exist which work well with the LAMP Platform. Instructions for setting up the Platform on 3rd party cloud resources are not available, as the use of Docker Swarm with the Traefik router software is recommended. When used in the supported configuration, regions of compute resources are segregated into Production or Staging, and tagged with the appropriate CPU, RAM, Disk I/O, and Network I/O parameters such that Docker Swarm may automatically distribute Stacks to the correct sub-clusters which are configured and maintained internally. The Traefik router software interfaces with Docker Swarm and the configured DNS service to provide external and internal access to services according to the configuration files in the GitHub repository. For staging and development purposes (primarily data analysis), JupyterLab and RStudio are provided in Stack files for quick localized deployment such that data access incurs the lowest possible bandwidth and latency cost.

THE AUTOMATIONS FRAMEWORK

Automations are a flexible framework for the LAMP platform that allow you to run complex analytics and decision support tools either in reaction to new events in an event stream, or on a periodic schedule. Without having to configure a processing pipeline for system requirements such as CPU, I/O, or RAM, automations abstract the functional logic from data resources and system requirements. Automations support simple, flexible, and portable code that can run on low-power devices such as smartwatches or older smartphones all the way up to large servers and computing clusters in the cloud.

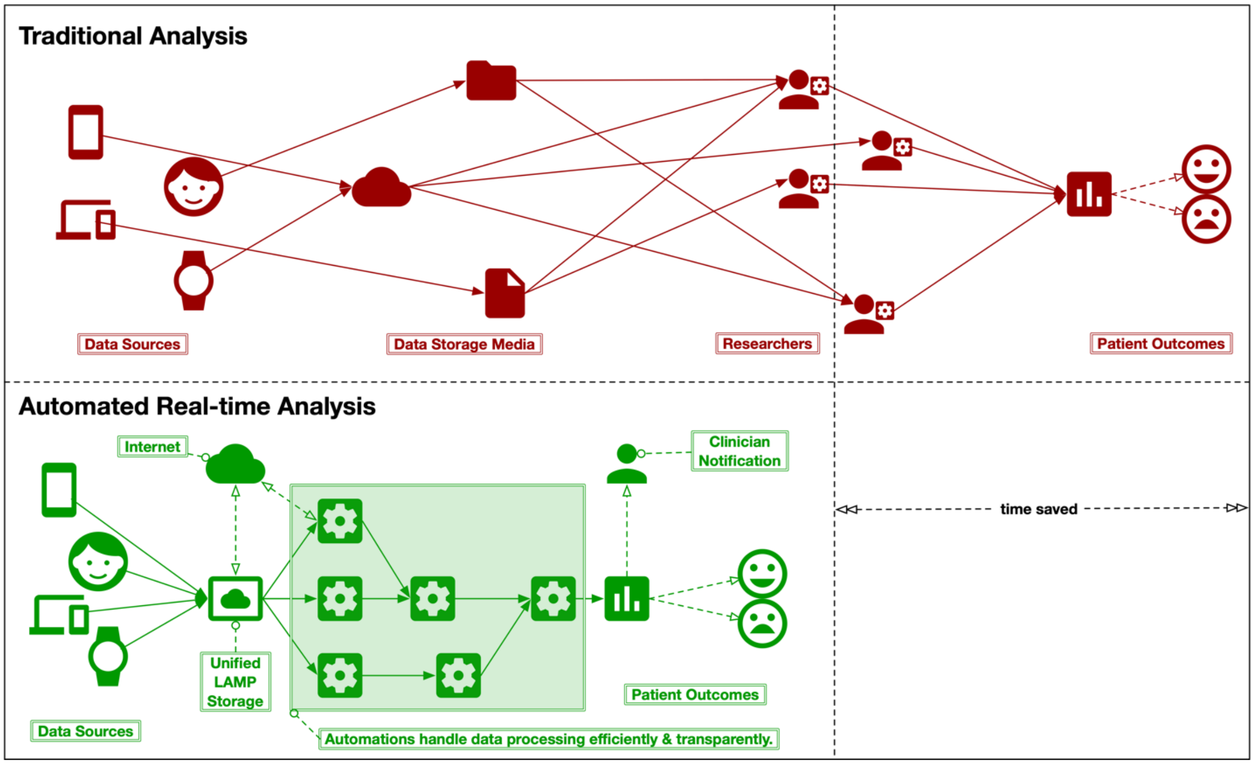

These "applets," called Automations, can be written in typical data science programming languages such as JavaScript, Python, and R, with any packages or dependencies automatically bundled within. When installed onto a Resource (that is, a Researcher, Study, Participant, or even an Activity), it is capable of listening to events generated by that Resource. For example, if installed for Participants, one such applet could listen to any SensorEvents or ActivityEvents, or when installed for Activities, it could listen only to anonymized ActivityEvents generated by any Participant. When the Cloud server receives new events, it prepares all Automations that fit the specified event mask and allows them to execute with preallocated hardware limits.

Tags as Arbitrary Data on Resources

Tags are an arbitrary unit of extensibility available to all Resource sub-types. Through string-indexed/keyed subscripting, out-of-line data may be attached to objects in the LAMP Platform as an ad-hoc micro-database. For a flow chart on the usage of Tags, see the figure below. Tags are a powerful tool that may be leveraged by clients of the LAMP Platform to build applets for the Platform as well as smaller apps within such client apps themselves.

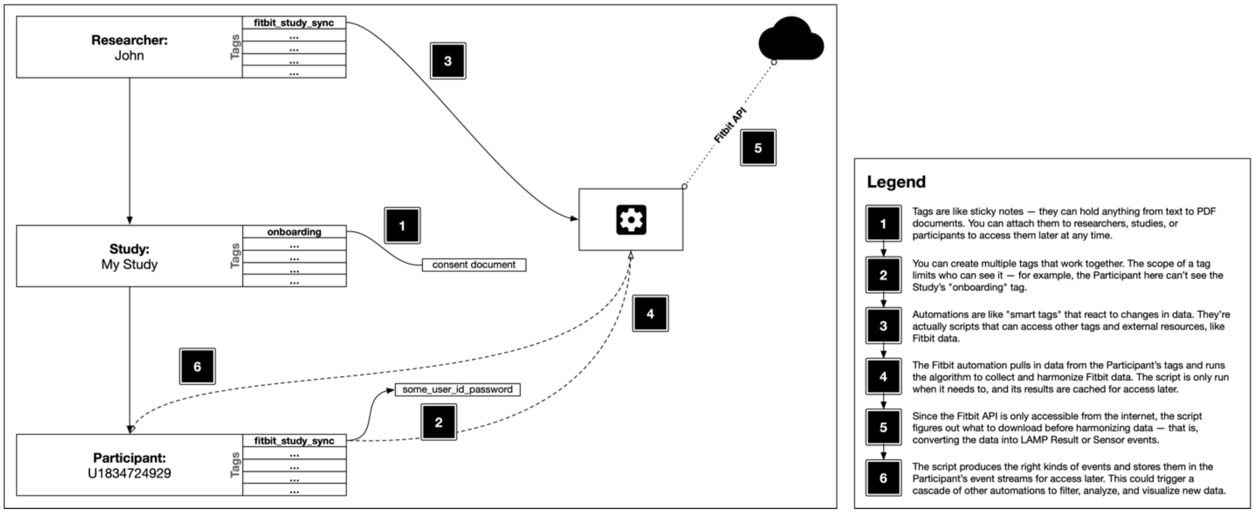

Data-URI strings in Tags

Tags may consist of JSON object, array, or primitive types, as well as encoded data-uri strings. Data-uri strings are normal string primitives but prefixed with "data:<mime_type>[;base64]," where "<mime_type>" refers to the binary application file type of the data that follows, such as "application/json", "text/plain; charset=utf8", or "image/svg+xml". If the "base64" optional parameter is provided, the contents of the string following the comma are to be base64-decoded when interpreted by the LAMP Platform or clients of the LAMP Protocol. Specifying an optional "Accept" header type may optionally allow the LAMP server component or other LAMP Protocol vendors to automatically convert such data-uri strings into a binary type.

Atomic Indexed Access and Modification

Furthermore, to support atomic operations on Tags, an indexed modifier version of get & set methods shall exist such that for a Tag whose content is an object, the method "GET | POST /type/<id>/my.tag.name.here[/someKeyedIndex]" shall return or replace only the sub-content of the object but not the whole object represented by the Tag. For JSON arrays, keyed indices shall take the form of continuous numbered indices found in the array itself, including the special index "length" which shall only return but not replace the length of the underlying array. Through these rudimentary atomic mutation facilities, vendors and clients of the LAMP Protocol may perform basic synchronization without poll-waiting or SSE (Server Sent Events) reconciliation.

Automations as Multidimensional Planes of Data within Tags

Automations shall be represented by their specific LAMP Protocol object schema, but encoded as a plaintext JSON data-uri string with the mime type "application/vnd+lamp.automation". When registering or unregistering an Automation's availability with a LAMP server or other component, the component itself shall maintain a running record of compute images, trigger-points, and code for each Automation. When the Tag containing the Automation data is removed, the Automation itself shall be unregistered and made no longer functional in that instance of the LAMP Platform. The figure below describes the relationship between the static data plan (Tags) and the dynamic data plane (Automations) which leverage the functionality described in prior chapters to perform Just-In-Time intervention, prediction, analysis, visualization, or some other set of relevant functions.

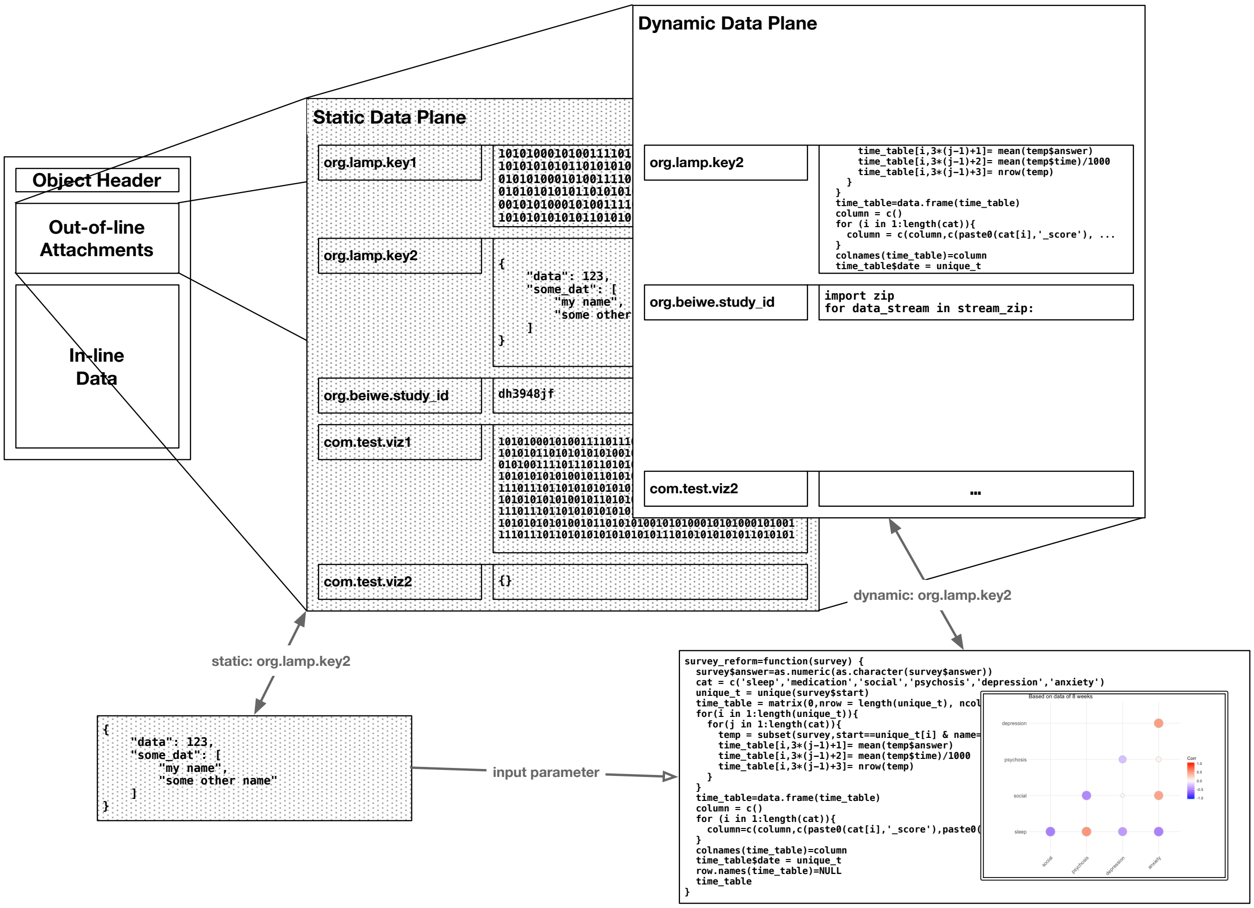

Federated Systems Using the Automation Framework

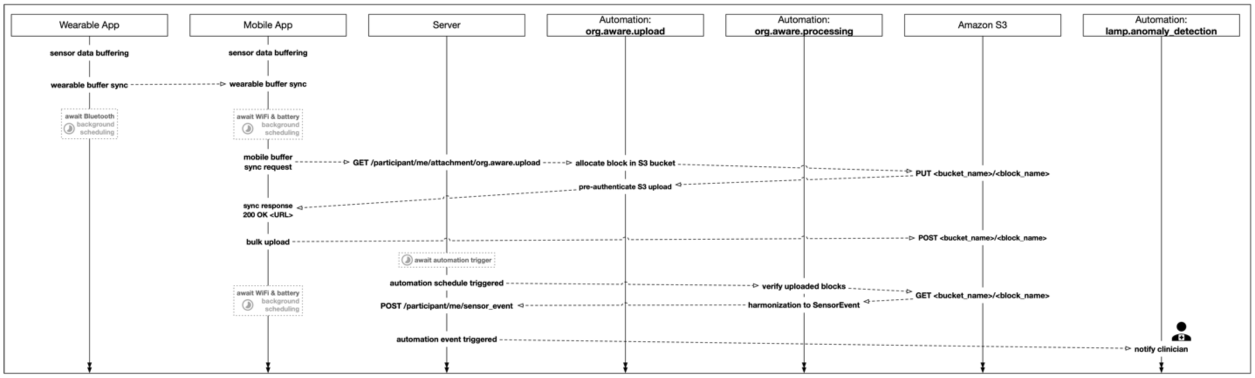

Supposing multiple existing systems provided clinically useful sources of data, such as longitudinal imaging repositories or existing Fitbit devices synchronized to the cloud. While data retrieval and ad-hoc storage of "out-of-line" (that is, unrecognized by the Platform, but retaining meaning to its owner) data from within the Platform is simple using the API, it would be simply infeasible to manually verify modified data against multiple specific conditions and run several scripts in the Researcher's local computer before sending out notifications or awaiting further processing from elsewhere. Instead, the Platform supports, through the Automations framework, a method of dynamically running such scripts as "applets" atop extremely powerful unconstrained hardware not managed by the Researcher or their IT department.

In the example above, a combination of two applets and an external Amazon S3 database (unknown to the LAMP Platform) provide the equivalent three step upload-process-analyze functionality of apps such as AWARE, Fitbit, Beiwe, Google Fit, and more. The "lamp.anomaly_detection" applet is not considered a part of this group as it was written to use only the standard API provided by the LAMP Platform; it contains no knowledge of the other two applets and the external database. The "org.aware.upload" applet requests preallocation of storage, perhaps on the order of ~5GB, but entirely variable depending on the Participant's device or historical data uploads. It then returns a response immediately to the requesting smartphone device or internet service with a URL to which it can upload the data. The second applet, "org.aware.processing" is instead run by the Cloud server every 5 minutes to check if any processing needs to be done in the database, and if so, executes the processing, but otherwise does nothing. This applet converts the uploaded data to LAMP Resources (ActivityEvents or SensorEvents, specifically) and submits them to the Cloud server in bulk. Just as with any other events received by the Cloud server, it will then execute a set of Automation applets — in this case, "lamp.anomaly_detection." In summary, with this multi-applet workflow, data is automatically uploaded and stored in an external database wholly maintained by a third-party, subsequently converted to actionable reactive LAMP Sensor or ActivityEvents, and finally analyzed through the same methods as all other data.
